# A Comprehensive Overview of Antimicrobial Peptides: Broad-Spectrum Activity, Computational Approaches, and Applications

**DOI:** 10.3390/antibiotics14111115

**Published:** 2025-11-05

**Authors:** Camila Langer Marciano, João Vítor Félix de Lima, Murilo Sousa do Couto Rosa, Rafaelly Avelar do Nascimento, Antonio de Oliveira Ferraz, Iago Castro da Silva, Taís Nader Chrysostomo-Massaro, Nathália Gonsales da Rosa-Garzon, Hamilton Cabral

**Affiliations:** 1 Department of Pharmaceutical Sciences, School of Pharmaceutical Sciences of Ribeirão Preto, University of São Paulo, Ribeirão Preto 14040-903, Brazil; 2 Department of Biology, Faculty of Philosophy, Sciences and Letters at Ribeirão Preto, University of São Paulo, Ribeirão Preto 14040-901, Brazil

**Keywords:** bioactive peptide, food preservation, machine learning, peptide database

## Abstract

Among bioactive peptides, those with antimicrobial activity have attracted increasing attention due to their potential as alternatives to traditional antibiotics. Antimicrobial peptides (AMPs) are small molecules, typically composed of 6 to 60 amino acid residues, and some with low cytotoxicity and minimal side effects. They exhibit broad-spectrum activity against bacteria, viruses, fungi, and parasites through diverse mechanisms of action and interactions with the immune system. This review presents the main aspects of AMPs, including their biochemical characteristics, sources, mechanisms of action, and computational tools used for their identification and analysis. It also examines recent progress in clinical trials and the current limitations that restrict the development and commercialization of AMPs. The review discusses the application of AMPs beyond human medicine, including their use in food preservation to prevent microbial contamination and in veterinary medicine to control infections in livestock, thereby reducing dependence on conventional antibiotics. Overall, AMPs represent a versatile class of antimicrobial agents whose effective implementation across health, food, and veterinary sectors will require integrated multidisciplinary approaches.

## 1. Introduction

Bioactive peptides (BAPs) are small molecules that usually contain 2–50 amino acid residues and have molecular masses less than 10 kDa [1]. However, BAPs with longer chains, consisting of up to 50 amino acids, have also been reported, likely due to improvements in peptide synthesis and manufacturing technologies [2]. The biological activity of peptides depends on their amino acid composition and sequence, which determine their physiological effects. BAPs have been gaining increasing attention in biomedical, food, and pharmaceutical sciences because of their specific functional properties, such as antimicrobial, antioxidant, immunomodulatory, and antitumor activities [3,4]. These amino acid oligomers are present in a wide variety of organisms and play key roles in cellular communication, immune defense, and metabolic regulation [3]. Although numerous studies have reported various potential biological activities of peptides, their antimicrobial potential remains highly promising [5].

Antimicrobial peptides (AMPs) are molecules capable of combating disease-causing microorganisms such as bacteria, viruses, fungi, and parasites through different mechanisms. The first report on the identification of AMPs was published in 1939 by René Dubos, who isolated a compound (gramicidin) from a *Bacillus* lineage that could protect mice against pneumococcal infections [6]. Subsequently, a series of AMPs have been identified in a wide range of organisms, which employ them in defense strategies against pathogens [7]. The Antimicrobial Peptide Database 3 (APD3), which was updated in September 2025, currently includes data on 5680 peptides. Of this, 3351 are natural AMPs, 1733 synthetic, and 329 predicted. In terms of biological activity, 4865 peptides exhibit antibacterial effects, 1664 antifungal, 348 antiparasitic, and 270 antivirals [8].

Most AMPs consist of 6 to 60 amino acid residues and typically exhibit a positive net charge and cationic properties [9]. Unlike conventional antibiotics, AMPs kill microbial cells by disrupting membrane integrity or the ionic gradient through interactions with the negatively charged cellular membrane. They have multiple mechanisms of action, such as inhibiting the synthesis of proteins, DNA, and RNA, or interacting with specific intracellular targets [10,11].

## 2. Broad-Spectrum Activity of Peptides

**Figure 1:**
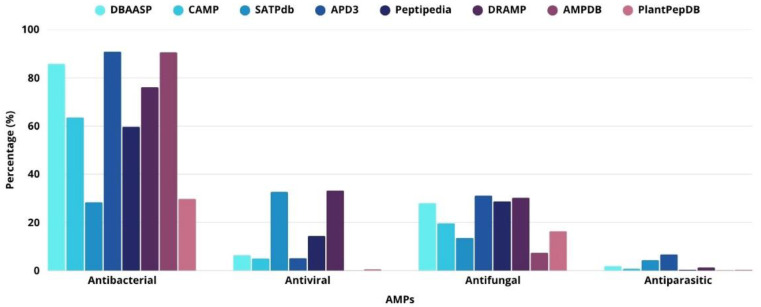
Distribution of AMPs according to their specific activities in different databases. Information obtained from databases available at: DBAASP (https://dbaasp.org/home) [12]; CAMP (https://camp.bicnirrh.res.in/) [13]; SATPdb (http://crdd.osdd.net/raghava/satpdb/) [14]; APD3 (https://aps.unmc.edu/AP/) [8]; Peptipedia (https://app.peptipedia.cl/) [15]; DRAMP (http://dramp.cpu-bioinfor.org) [16]; AMPDB (https://bblserver.org.in/ampdb/) [17]; PlantPepDB (http://www.nipgr.ac.in/PlantPepDB/) [18] (accessed on 24 August 2025).

Antimicrobial peptides can act against different microorganisms, and several databases compiling various aspects related to AMPs have been established. Figure 1 shows the types of activities associated with AMPs: antibacterial, antiviral, antifungal, and antiparasitic activities.

### 2.1. Antibacterial Activity

Most AMPs have been reported to show antibacterial effects. This may be related to the large number of studies seeking approaches to combat antibiotic resistance (AMR), a major challenge to global public health that has resulted in hard-to-treat infections and a shortage of effective therapies [19,20]. Moreover, the lack of effective actions to control these microorganisms can have devastating consequences, claiming millions of lives and increasing treatment costs to over USD 100 trillion annually [21]. The organization of pathogenic microorganisms into biofilms prevents antibiotics from interacting with the bacterial cells [22]. Peptides have also been explored as potential solutions to counteract the aggravating factors in AMR [23].

According to their mechanism of action, antibacterial peptides can be classified as membrane peptides (e.g., those that affect the structure of the plasma membrane) and non-membrane peptides (e.g., those that affect the synthesis of nucleic acids and proteins). In addition to their effect on microorganisms, some AMPs are immunomodulatory and help combat infectious processes. Therefore, these AMPs have limited potential for enhancing resistance, as they target highly conserved structures [7].

### 2.2. Antiviral Activity

Despite the predominance of AMPs combating bacterial infections, AMPs targeting viral infections have gained importance because of the high number of deaths caused by these infections. This has led the World Health Organization (WHO) to develop a list of priority diseases to accelerate research and development. In July 2024, the list included 28 viral families, and the priority diseases, which are associated with a higher risk of epidemics or pandemics, included Ebola virus disease, Marburg virus disease, Lassa fever, Crimean-Congo hemorrhagic fever, Middle East respiratory syndrome (MERS), severe acute respiratory syndrome (SARS), other known viral diseases, and the still-unknown “Disease X.” Concerns regarding the risks associated with these diseases are reported monthly by the WHO, with records showing that they affect various regions of the world [24].

In addition to mortality rates, the emergence of new viruses, viral resistance mechanisms, delays in vaccine development, and the severe adverse effects of antiviral drugs have contributed to the complexity of this problem. In this context, natural and synthetic antiviral peptides (AVPs) have emerged as a new class of antiviral agents offering several advantages [25].

### 2.3. Antifungal Activity

The growing incidence of fungal infections has become a topic of concern since millions of individuals are estimated to die annually as a result of such infections. Some of the main human fungal pathogens include *Candida albicans*, *Cryptococcus neoformans*, *Aspergillus fumigatus*, and the emerging multidrug-resistant *Candida auris*. The increase in fungal infections is associated with several factors. When considering the currently available treatment options, the limited number of antifungal drug classes combined with the development of resistance across different genera has driven the search for integrated and effective strategies [26].

Another factor that favors fungal infections is the growing number of vulnerable individuals resulting from various treatments involving immunosuppressive and chemotherapeutic drugs. In this context, antifungal peptides (AFPs) possess properties that could help overcome the limitations imposed by current therapies and can be applied to the control of fungal contamination in food products [27].

### 2.4. Antiparasitic Activity

Among the AMPs identified in various databases, those with antiparasitic activity were the least frequently represented. Parasitism is a biological interaction that can lead to disease and, in severe cases, result in host death. Among the most relevant human parasites are protozoans (e.g., *Toxoplasma gondii*, *Plasmodium* species, and *Trypanosoma cruzi*), which cause infections classified by the WHO as neglected diseases and, consequently, receive little investment for the development of new treatments. For some groups, the available drugs are toxic and only partially effective, and the emergence of resistant strains has driven the search for alternative strategies [28]. In this scenario, recent advances in research on antiparasitic peptides have demonstrated that these molecules can act through specific mechanisms and targets and may, therefore, represent innovative solutions for combating these pathogens [29]. The peptides cruzioseptin-1, cruzioseptin-4, cruzioseptin-16, dermaseptin-SP2, and pictuseptin-1 (originally identified in frog skin secretions) demonstrated in vitro cytotoxic activity against *Leishmania mexicana*, *Plasmodium falciparum*, and *Trypanosoma cruzi,* while showing low toxicity in human cell lines [30].

## 3. Approaches for Obtaining AMPs

With advances in biotechnology and protein engineering, the demand for efficient, scalable, and selective peptide production methods has substantially increased. Different routes are employed to produce peptides of interest, including chemical synthesis, enzymatic hydrolysis of proteins, extraction from natural sources, and expression through recombinant technologies. Each method shows specific advantages and limitations in terms of cost, purity, yield, stability, specificity, scalability, and the possibility of structural modifications (Table 1) [31,32].

### 3.1. Peptide Production via Chemical Synthesis

Chemical peptide synthesis is one of the most widely used approaches for producing specific peptide chains, enabling the precise modification of amino acid sequences with high reproducibility and structural control. This method is particularly important in the industrial and pharmaceutical contexts, where the production of peptides with well-defined characteristics (such as stability, enzymatic resistance, or optimized biological activity) is essential [4]. One of the major advantages of this approach is the possibility of incorporating non-natural amino acids or artificial post-translational modifications such as cyclization, glycosylation, and even fluorescent or radioactive labeling, which significantly broadens the applications of the peptides in pharmacokinetic and therapeutic studies [32].

The classic solid-phase peptide synthesis (SPPS) method remains the most widely adopted method, primarily because of its compatibility with automated and scalable processes. In this process, peptide assembly begins at the carboxyl end, which is anchored to a solid resin. Protected amino acids are added individually in alternating steps of coupling and deprotection until the full peptide chain is built. In some cases, hybrid solid- and liquid-phase synthesis can be employed, combining the stepwise assembly control of solid-phase synthesis with the chemical flexibility of liquid-phase methods, thus enabling the efficient production of longer or structurally complex peptide chains [33].

The choice of coupling reagents, such as O-benzotriazole-N,N,N′,N′- tetramethyl-uronium-hexafluoro-phosphate (HBTU), O-(7-azabenzotriazol-1-yl)-N,N,N,N′ -tetrame thyluronium hexafluorophosphate (HATU), or N,N′- diisopropylcarbodiimide (DIC), as well as protection systems (e.g., Fmoc–9-fluorenylmethyloxycarbonyl and Boc-tert-butyloxycarbonyl) directly influences the reaction efficiency, final purity, and failure rates during synthesis [3].

In certain situations, hybrid approaches combining solid- and liquid-phase syntheses are used to produce larger peptides or peptides with more complex cyclic structures. A well-known example is cyclotide kalata B1, a cysteine-rich peptide found in plants and characterized by a cyclic cystine knot (CCK) motif. Kalata B1 shows strong antimicrobial properties and is being studied as a structural framework for the development of therapeutic peptides, including receptor agonists and enzyme inhibitors [32].

Despite its versatility, chemical synthesis faces major challenges in scaling up the production of long or structurally intricate peptides in a cost-effective manner. Additionally, traces of leftover organic solvents and toxic reagents, such as dimethylformamide (DMF) and carbodiimide-based activators, require thorough purification, which can substantially increase production costs [4].

### 3.2. Peptide Production via Enzymatic Pathways

The production of peptides through enzymatic hydrolysis is a widely explored alternative, especially when seeking a more sustainable process with lower environmental impact and greater cleavage selectivity. This approach is based on the controlled action of proteases on natural proteins derived from milk, algae, eggs, fish, or plants, generating BAP fragments with various functions such as antioxidants, antihypertensive, antimicrobial, and other activities [4,33].

Bioactive peptides generated through enzymatic hydrolysis are often referred to as cryptopeptides or cryptic peptides, as they remain inactive within the native structure of their parent proteins and are only released following specific cleavage. These sequences are typically hidden within the full-length protein and become biologically active only under controlled hydrolytic conditions, supporting the concept that proteins act as reservoirs of latent peptides with distinct physiological effects [33,34]. The choice of enzymes, such as trypsin, chymotrypsin, pepsin, alcalase, flavourzyme, or microbial proteases, depends on the desired cleavage pattern and the nature of the protein source. Advances in enzymatic processing, including both in vitro and in silico approaches, have enabled the identification and characterization of these encrypted peptides [4]. For example, Bosso et al. [35] identified a cryptic peptide located at the C-terminal region of casein. This peptide, named KNR50, was recombinantly expressed and demonstrated antimicrobial activity both in vitro and in vivo. Notably, the authors highlight that its mechanism of action minimizes the risk of resistance development [35].

Recent technological developments have facilitated the combination of physical techniques with enzymatic hydrolysis to boost both the yield and specificity of peptides. Pretreatments such as ultrasound-assisted extraction (UAE), pulsed electric fields (PEF), and high hydrostatic pressure (HHP) can help improve enzyme access to the inner regions of the protein, increase the efficiency of hydrolysis, and enable the production of peptides with specific bioactivities [33].

For instance, enzymatic hydrolysis of marine microalgae such as *Tetraselmis suecica* has been shown to produce peptides with strong antimicrobial properties. One variant, AQ-1766, demonstrated significant efficacy against a wide range of pathogenic bacteria, including *Escherichia coli*, *Staphylococcus aureus*, *Salmonella typhimurium*, *Listeria monocytogenes*, and *Pseudomonas aeruginosa*. These peptides typically disrupt microbial membranes and have potential in food preservation and therapeutic applications [34].

Enzymatic processes offer several advantages, including high specificity, environmental compatibility, and production of peptides that are generally considered safe, often eliminating the need for further chemical modifications. Nonetheless, this approach presents challenges such as variability in raw materials, high enzyme costs, and the need for additional purification steps, such as ultrafiltration, chromatography, and lyophilization [4,34].

### 3.3. Peptide Extraction from Natural Sources

Direct extraction of peptides from natural sources is one of the oldest methods still in use today, especially for AMPs and immunomodulatory compounds. A wide range of organisms, including plants, marine animals, bacteria, and fungi, naturally produce BAPs with important biological roles in pathogen defense, cell signaling, and metabolic regulation [1,3].

In plants, many of these defense-related peptides are rich in cysteine and are stabilized by disulfide bonds, which help maintain their three-dimensional structures. Well-known peptide families exhibiting potent antifungal and antibacterial activities, such as defensins, cyclotides, thionins, and snakins, can be isolated from plant tissues such as leaves, seeds, and roots using conventional extraction methods such as maceration, solvent precipitation, chromatography, and ultrafiltration. Other examples include lipid transfer proteins (LTPs) and hevein-like peptides, which also contribute to pathogen resistance and can be extracted through similar procedures [31,32]. The antimicrobial activity of these molecules is generally associated with their cationic nature and their interactions with anionically charged membranes of microorganisms [32].

Antimicrobial peptides have been extensively described in marine organisms such as sponges, mollusks, echinoderms, and fish. These molecules include arenicins from marine worms, piscidins from fish, and holothuroidins from sea cucumbers, which inhibit the growth of multidrug-resistant bacteria, fungi, and viruses even in extreme environments. Other noteworthy examples include mytilins from mussels and defensin-like peptides from marine sponges, both of which display broad antimicrobial spectra [36,37,38]. Extraction of these molecules involves isolation from tissues or fluids (such as mucus, hemolymph, or pseudocoelom), followed by purification using reversed-phase HPLC, size-exclusion or ion-exchange chromatography, and subsequent structural analysis by mass spectrometry and NMR spectroscopy [3].

Microorganisms such as bacteria and fungi produce AMPs as part of their defense mechanisms. Nisin, produced by *Lactococcus lactis*, is a well-established example, widely used as a food preservative (E234) due to its strong activity against Gram-positive bacteria [39]. Additionally, one of the first defensins isolated from fungi was plectasin, obtained from *Pseudoplectania nigrella* and acts by inhibiting bacterial cell wall biosynthesis through binding to a cell wall precursor [40]. Other microbial AMPs, such as subtilin (*Bacillus subtilis*), lacticin 3147 (*Lactococcus lactis*), microcin J25 (*Escherichia coli*), and thuricin CD (*Bacillus thuringiensis*), exhibit antimicrobial properties, with potential applications in food preservation and medical therapy [7,39,41,42].

Despite the high therapeutic potential and natural diversity of these peptides, direct extraction from natural sources faces major limitations such as low yields, seasonality, and complexity of the extracts; these factors limit the ability to obtain such compounds on an industrial scale. Therefore, many natural peptides are cloned or synthesized using recombinant or chemical methods once their sequences have been identified [33,34].

### 3.4. Peptide Production via Recombinant Technology

Recombinant DNA technology is widely used for large-scale peptide production, especially for peptides that cannot be feasibly obtained using natural extraction or traditional chemical synthesis. In this approach, genes encoding the peptides of interest are inserted into plasmid vectors and expressed in hosts, such as bacteria, yeast, filamentous fungi, or even mammalian cells [27].

Recombinant production is particularly advantageous for producing peptides with specific post-translational modifications or when utilizing cellular systems for proper folding and disulfide bridge formation, as in the case of cyclotides and defensins. For small peptides, techniques such as fusion expression with carrier proteins (e.g., glutathione S-transferase [GST] or maltose-binding protein [MBP]) are used to increase solubility and facilitate purification [43].

The recombinant production of bacteriocins using genetically modified *Lactobacillus* strains allows the use of GRAS (generally recognized as safe) organisms to produce bioactive compounds for functional and therapeutic food applications. Common GRAS species include *Lactobacillus plantarum*, *L. casei*, *L. rhamnosus*, and *L. acidophilus* widely accepted due to their long history of safe use in fermented foods and probiotic products [44]. Other frequently used GRAS organisms in food biotechnology include species from the genera *Bacillus* (e.g., *B. subtilis*, *B. coagulans*), *Streptococcus thermophilus*, and the yeast *Saccharomyces cerevisiae* [45]. Other strategies, such as the use of strong promoters, site-directed mutagenesis, and codon optimization, have also been explored to enhance recombinant peptide yield and expression efficiency [33,46].

The main challenges of this approach include peptide toxicity to the host organism, formation of inclusion bodies, and the need for additional purification steps. Despite these challenges, recombinant methods are considered one of the most promising methods for producing BAPs with high purity and relatively low cost, particularly for pharmaceutical and industrial applications [27,32].

## 4. Biochemical Characteristics of AMPs

**Figure 2:**
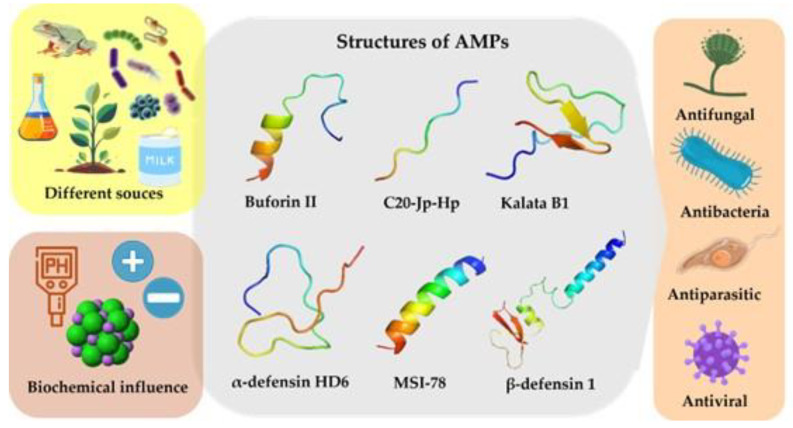
The scheme represents the different sources of production, biological activities and biochemical influences of AMPs. The central part shows examples of structures described for AMPs. Information obtained from DRAMP (http://dramp.cpu-bioinfor.org/) [16] and predicted by PEP-FOLD3 (https://bioserv.rpbs.univ-paris-diderot.fr/index.html) [47] (accessed 25 August 2025) and figures constructed with clean png (https://www.cleanpng.com/).

Antimicrobial peptides come from various sources, and their structural and biochemical characteristics, such as amino acid composition and three-dimensional structure, affect both their biological activity and mechanism of action (Figure 2). Despite their diversity, many AMPs share common features, including low molecular weight, positive charge, and hydrophobic residues [5].

### 4.1. Structure

The most common secondary structures in AMPs are α-helices, as seen in melittin, (from bee venom) [48], and β-sheets, as in ramoplanin (from *Actinoplanes*) [49]. Other structure types include linear peptides, such as the synthetic Bac8c [50], and cyclic peptides stabilized by disulfide bridges, as seen in novexatin (synthetic) [51,52]. Plant-derived peptides show specific conformations due to the rich content of amino acids such as thionine and cysteine, which not only favor antifungal and antimicrobial activity but also enable functions like ribosome inactivation and trypsin inhibition [31]. For example, cyclotides which show a three-dimensional structure containing loops and disulfide bridges, exhibit favorable biological activity and show resistance to proteolytic enzymes [53]. The combination of α-helix and β chains is an important condition for antimicrobial activity, as observed in human β-defensin 1 [54]. While α-helix favors membrane ruptures, β-sheets and cyclical structures are often involved in peptide internalization [36]. Additionally, some tryptophan-rich AMPs can show transmembrane effects, promote ion channel formation and lead to rupture [55].

### 4.2. Charge, pH, Saline Concentration

For antimicrobial function, peptides with a positive charge under physiological conditions and amphipathic chains are more common, as shown in Table 2. According to APD3, 87% of active peptides are cationic, 7% are neutral, and 6% are negative [8]. The positive charge interacts better with the anionic membranes of pathogens, promoting better interactions in the destruction and lysis capacity. Most AMPs have net charges ranging from +2 to +11, due to a high content of lysine and/or arginine residues [56,57].

Anionic AMPs have a net charge ranging from −1 to −8 and mostly generated as peptide fragments through proteolysis. These peptides interact with microbial membranes by using metal ions and negatively charged components to form salt bridges, such as the synthetic peptide glutoxim [62,92]. Some AMPs display both anionic and cationic regions, such as F8 from egg yolk. They are typically derived from larger precursor proteins and exhibit antimicrobial activity by interfering with cell division and targeting the cell poles [92,93].

Furthermore, the pH and saline concentration can also affect the antimicrobial potential of highly active structures. High salinity is a determinant of peptide activity. Most structures are stable in the pH range of 4 to 6 because an acidic pH favors the protonation of some amino acids and the action of positive charges against the bacterial cell wall [94].

### 4.3. Hydrophobicity

The hydrophobicity of AMPs modulates their efficiency and specificity. Most AMPs contain approximately 50% hydrophobic residues. This favors the entry of these peptides into the lipid bilayer of pathogens [57]. There is no perfect combination of positive charge and hydrophobic chain length that can maximize activity and reduce cytotoxicity, instead the balance between positive charges and hydrophobicity modulates the interaction of the peptide with microorganisms. Higher hydrophobicity at a given charge has been shown to increase the antimicrobial effect, whereas lower hydrophobicity decreases this effect [56]. However, this relationship is not always proportional, and reports have shown that increments in hydrophobicity can reduce antimicrobial activity and toxic effects in mammals [57]. Therefore, the ratio of charge to hydrophobicity is a key factor in the Pasupuleti development of AMPs. Brogden [9], proposed that ratios ranging from 1:1 to 2:1 between the hydrophobic and basic residues may be an efficient combination. Thus, amphipathic chain characteristics and positive charges can improve the efficiency of antimicrobial activity [9,95].

## 5. Antimicrobial Peptide Action Mechanism

The amino acid sequence, hydrophobicity, charge, and three-dimensional structure are essential for understanding the mode of action of AMPs. Variations in these aspects across AMP classes ensure specific interactions with their respective targets. AMPs can interact directly with the target microorganism (cell surface or intracellular components), leading to its death, or act indirectly by modulating the host immune system to combat the infection. In addition, many AMPs possess the ability to act on multiple targets and, consequently, exert more than one convergent mechanism of action contributing to their antimicrobial activity [92,96].

### 5.1. Extracellular Target AMPs

Several factors influence the ability of AMPs to permeate and destabilize microbial membranes, such as amino acid sequence, membrane lipids, and peptide concentration. The lipid composition of cell membranes plays a crucial role in the selectivity and effectiveness of antimicrobial peptides. Bacterial membranes are negatively charged due to the presence of phospholipid head groups such as phosphatidylglycerol (PG), cardiolipin (CL), or phosphatidylserine (PS). Additionally, the presence of lipopolysaccharides (LPS) in Gram-negative bacteria and teichoic acids in Gram-positive bacteria contributes to this negative charge [97,98]. These compositional differences between prokaryotic and eukaryotic membranes are important to the selective action of AMPs. The physical properties of membranes, such as fluidity, tension, and lipid packing, also influence AMP interactions, making both lipid composition and membrane mechanics critical factors for peptide efficacy and selectivity [99,100].

In addition to anionic phospholipids, molecules such as cholesterol and sphingolipids are important in maintaining membrane stability and fluidity, directly influencing interactions with antimicrobial peptides. In eukaryotes, cholesterol acts as a permeability regulator, reducing the space between lipid chains and hindering the insertion of AMPs into the lipid bilayer. As a result, mammalian membranes, rich in cholesterol, are generally more resistant to AMP-induced lysis, unlike bacterial membranes, which contain lower levels of cholesterol [101]. Additionally, studies have shown that pore formation is strongly influenced by lipid composition, as well as by the bilayer’s mechanical and structural properties, with cholesterol being a major factor in enhancing membrane stability and resistance to external stress [99,101].

Membrane-active AMPs are typically amphipathic and positively charged at physiological pH 7.4. Due to their net positive charge and hydrophobicity, AMPs interact with negatively charged bacterial membranes, where the negative charge arises from lipid head groups and other anionic membrane components. This interaction is mediated by electrostatic forces on the bacterial surface, altering the cell’s electrochemical potential, disrupting lipid organization, and leading to leakage of intracellular contents [93,102].

In general, AMPs follow two major types of membrane action: pore formation (barrel-stave and toroidal pore) and non-pore mechanisms (carpet model and detergent-like action) [93,102]. In the Barrel-stave model, AMPs insert perpendicularly into the lipid bilayer, assemble into oligomers that form transmembrane pore with a hydrophilic interior [96]. Hydrophobic residues interact with the fatty acid chains within the membrane, stabilizing pore formation [98]. The synthetic peptide Mo-CBP3-Pep-CPAIQRCC and magainin2 (GIGKFLHAS KKFGKAFVGEIMNS, Frog) act via barrel-stave model due to their hydrophobicity and α-helical structure [48,103]. In the Toroidal pore model, peptides interact with the polar head groups of membrane lipids to form pores, causing the lipid bilayer to bend around the peptide and form a continuous curvature, resulting in stable channels that allow the passage of ions and other molecules [97,104]. Peptides such as YK5 (NKVKEWIKYLKSLFK, synthetic) and MSI-78 (GIGKFLKKAKKFGKAFVKILKK, synthetic) exemplify this mechanism by forming toroidal pores in membranes [97,104].

In the Carpet model, peptides accumulate on the membrane surface at high concentrations, aligning parallel to the membrane to form a dense layer resembling a carpet. When a threshold concentration is reached, this layer disrupts and lyses the membrane without forming pores [98,105]. Cecropin P1 (SWLSKTAKKLE NSAKKRISEGIAIAIQGGPR, nematode) acts via this model, coating the lipid bilayer and inducing membrane rupture [106]. In the Detergent-like model, AMPs disrupt the membrane by solubilizing lipids, leading to micelle formation and complete bilayer disintegration. This results in dissipation of the electrochemical gradient. Although structurally predisposed to pore formation, the peptide PepD2M-Myr (WKKLKKLLKKLKKL-NH2, synthetic) acts via a detergent-like mechanism, collapsing the membrane into lipid aggregates [96].

Some AMPs act via alternative or combined mechanisms. The S-thanatin (GSKKPVPIIYCNRRSGKCQRM, insect) peptide exerts multiple actions, combining membrane depolarization and interference with the bacterial respiratory chain, while maintaining low toxicity to mammalian cells due to its selectivity for lipid composition [96]. Melimine (TLISWIKNKRKQRPRVSRRRRRRGGRRRR, synthetic) and Mel4 (KNKRKRRRRRRGGRRRR, synthetic) peptides, acting against *Pseudomonas aeruginosa*, follow a sequence of events including LPS neutralization, membrane depolarization, intracellular material release, and ultimately cell lysis [107]. The hRNase 7 peptide directly interacts with the OprI lipoprotein of *P. aeruginosa*, promoting increased membrane permeability and cellular content leakage [108]. Unlike the previously mentioned peptides, α-defensin HD6 (CLLQGRCNCNLKLGCKSGFCGGCVNLP, human) acts by forming nanonets on the bacterial surface without directly inducing cell death. This physical net traps bacteria and prevents adhesion and invasion, protecting the intestinal epithelium [109]. The F6 fraction from egg yolk hydrolysate contained three cationic and ten anionic peptides that may act in a synergistic manner. Cationic peptides destabilize bacterial membranes through electrostatic interactions, while anionic peptides may use metal ions to facilitate membrane penetration, suggesting that the overall effect of F6 results from the combined action of peptides with opposite charges [93].

Antifungal peptides have emerged as a promising alternative in combating fungal infections, especially in light of the global rise in resistance to conventional antifungals such as azoles, polyenes, and echinocandins [110]. The mechanism of action of AFPs involves the destabilization of the microbial cell membrane, a process mediated by electrostatic interactions between the cationic residues of the peptides and the anionic phospholipids of the membrane, such as phosphatidylglycerol. These peptides can create pores or disrupting membrane permeability, which compromises cellular structural integrity, therefore the result is cell death. Some examples include Mo-CBP3-Pep (CPAIQRCC, synthetic) and RcAlb-Pep (AKLIPTIAL, synthetic), which facilitate pore formation in fungal membranes and induce oxidative stress, thereby contributing to hyphal damage [111]. The AFP, derived from *Aspergillus giganteus*, interacts with the fungal membrane without forming pores but alters local membrane fluidity and compromises structural integrity, ultimately resulting in fungal cell destruction. Some antifungal peptides, such as CAADIVGQCPAKLK, derived from thaumatin, act through receptor-dependent mechanisms. This peptide interacts with the PHO36 receptor, found in *Saccharomyces cerevisiae* and *Candida albicans*, triggering apoptotic pathways mediated by oxidative stress [112].

The AVPs are strongly related to their physicochemical structure, which enables specific interactions with viral components or essential cellular elements involved in the viral replication cycle. Among these interactions, those with viral membranes, cellular receptors, or viral enzymes are particularly noteworthy worthy [113]. Inhibition of viral entry is one of the most common mechanisms, especially for enveloped viruses. Membrane active peptides display broad-spectrum antiviral properties by disrupting the stability of the viral envelope. According to Hoffmann [114], these molecules interact directly with the viral lipid bilayer, hindering endosomal fusion and inducing virion aggregation, effects reported for pathogens such as influenza, dengue, and herpes simplex viruses. Their specificity is linked to the pronounced curvature of viral membranes and to the lack of repair or homeostatic mechanisms in these structures. In vitro studies, the peptide p5 ([coil]-GGGYSKGGKGGGKGGKGGGKGGKGGGKGGKG, synthetic) prevents viral attachment to host cells by binding to a heparan sulfate proteoglycan (HSPG) located on the cell surface, thereby blocking the entry of human and murine cytomegaloviruses (HCMV and MCMV) [115]. Another example is enfuvirtide (Ac-YTSLIHSLIEESQNQQEKNEQELLELDKWASLWNWF-NH2, synthetic), which interferes with the formation of the six-helix bundle (6-HB) required for membrane fusion, while peptide C60 reinforces the pre-formed 6-HB, preventing further fusion events, particularly in endocytic pathways, with demonstrated efficacy against HIV [116]. In influenza A, inhibitory activity has also been observed for peptides such as C18 (ARLPR, synthetic) and C12-Hp (KKWK, synthetic) which target hemagglutinin subunits HA1 and HA2, respectively, blocking membrane fusion. Another peptide with a similar mode of action is C20-Jp-Hp (ARLPRKKWK, frog) which blocks the conformational rearrangement of HA2 and interferes with endosomal acidification, a crucial step for viral activation [117]. Peptides such as cecropin B (NH2-KWKVFKKIEKMGRNIRNGIVKAGPAIAVLGEAKAL-CONH2, cecropia moth), and CAP37 (NQGRHFCGGALIHARFVMTAASCFQ, human) have shown efficacy against non-enveloped viruses like adenovirus and rotavirus by promoting capsid disintegration or preventing viral entry through particle aggregation [118].

### 5.2. Intracellular Target AMPs

By crossing the cell membrane without the need to induce lysis, AMPs interact with intracellular components such as DNA, RNA, enzymes, and metabolic systems, ultimately leading to cell death. Peptides such as KT2 (NGVQPKYKWWKWWKKWW-NH2, synthetic), RT2 (NGVQPKYRWWRWWRRWW-NH2, synthetic), and PR-39 (RRRPRPPYLPRPRPPPFFPPRLPPRIPPGFPPRFPPRFP-NH_2_, pig) exemplify cell entry without pore formation or membrane lysis, a process facilitated by interactions with lipopolysaccharides and exploitation of the bacterial electrochemical gradient. Once inside the cell, they bind to DNA, thereby inhibiting transcription and translation [119,120]. Buforin II (TRSSRAGLQFPVGRVHRLLRK, *Asian Toad*) is also capable of translocating across the membrane without causing lysis and accumulate in the cytoplasm, where they strongly bind to bacterial DNA and RNA, resulting in inhibition of their functions [121,122]. Similarly, the AMP Oct-P2 (MFLVVKVLKYVV, octopus) interacts directly with plasmid DNA, promoting its aggregation and thereby impairing its functionality [123].

Some AMPs directly interfere with bacterial enzymatic activity. The peptide APKHKEMPFPKYP from bovine β-casein, acts against *Staphylococcus aureus* by inhibiting critical enzymes such as DNA gyrase and dihydrofolate reductase (DHFR) through hydrogen bonding, hydrophobic interactions, and π–π stacking. This interaction blocks DNA replication and cellular metabolism, ultimately leading to bacterial death [124]. The induction of reactive oxygen species (ROS) is a mechanism observed in several AMPs. The peptide LTX (315-KKWWKKW–β-difenílalanina–K-NH_2_, synthetic) induces oxidative stress by increasing the production of nitric oxide and hypochlorite, disrupting NAD+ metabolism and ATP production, and thereby compromising bacterial viability [125]. Peptides such as CM15 (KWKLFKKIGAVLKVL, synthetic) and MM63:CHx37 from bacteria, also induce oxidative stress by generating superoxide, hydroxyl radicals, and hydrogen peroxide, especially under aerobic conditions, while LL-37 (LLGDFFRKSFKKLGKRLLRFFLSKSA, human) causes oxidative stress by interfering with the cytochrome oxidase-bd complex in the periplasm [126]. The peptide [K4K15]-CZS-1 (GFLKIVKGVGKVALKAVSKLF, frog) not only permeabilizes membranes but also disrupts bacterial metabolism by inhibiting essential pathways such as fatty acid biosynthesis, phospholipid metabolism, and the tricarboxylic acid (TCA) cycle, thereby hindering cellular recovery and enhancing bacterial death [127].

In addition to cell membrane action of AFPs, many antifungal peptides target essential intracellular components related to fungal metabolism. AMPs such as histatin5 (DSHAKRHHGYKRKFHEKHHSHRGY, human) can interfere with genomic DNA without forming pores in the fungal membrane. As a result, these peptides cause morphogenetic defects, such as irregular chitin distribution, abnormal hyphal branching, and eventual cell lysis [128]. Metchnikowin (MQLNLGAIFLALLGVMATATSVLAEPHRHQGPIFDTRPSPFNPNQPRPGPIY) for example, derived from *Drosophila melanogaster*, acts specifically against *Fusarium graminearum* by inhibiting succinate-coenzyme Q reductase (SQR), an enzyme essential for mitochondrial energy production. Furthermore, Mtk interferes with fungal cell wall synthesis by inhibiting β (1,3)-glucanosyltransferase Gel1, a crucial enzyme for cell wall formation in *Fusarium* [129].

The peptide IJ4 (Ac-KW∆FWK∆FAK∆FAKNH2, shrimp) exhibits antifungal activity against various *Candida* species and other filamentous fungi by oxidative stress and stimulating the production of reactive oxygen species (ROS), which compromise fungal cellular components [130]. The peptide AMT (RRWWRF, synthetic) and its variants (AMT2- HRRWWRF, cyclo-AMT2, AMT3-HGHRRWWRF, and cyclo-AMT3) interact with fungal membranes, leading to structural disruption and membrane destabilization. These peptides were initially designed to inhibit lanosterol demethylase. However, studies indicated that their antifungal activity mainly relies on membrane interaction rather than enzyme inhibition [131].

The AVPs may also function through diverse pathways beyond extracellular binding. For example, the synthetic peptide WL-1 (GWKRIKQRIKDKLRNL) combats herpes simplex virus type 1 (HSV-1) by blocking viral attachment, suppressing replication, and downregulating pro-inflammatory cytokine production [118]. Direct inhibition of viral enzymes, such as influenza polymerase or essential replication proteases, has been observed for P9 (NGAICWGPCPTAFRQIGNCGHFKVRCCKIR, synthetic) and LVLQTM from β-casein [118].

### 5.3. Immunomodulatory AMPs

Antimicrobial peptides, also known as host defense peptides (HDPs), are produced by the immune system and play immunomodulatory roles that support pathogen clearance [132]. In humans, the main classes are defensins and cathelicidins, although other peptides, such as calprotectin and adrenomedullin, also contribute to immune responses [132,133]. HDPs regulate intracellular signaling pathways, including NF-κB, MAPK, and PI3K, by interacting with cytoplasmic proteins or G protein-coupled receptors. These interactions promote dendritic cell differentiation, leukocyte recruitment, and the production of anti-inflammatory cytokines, while inhibiting pro-inflammatory mediators such as TNF-α, IL-6, and IL-8. In addition, HDPs help bridge innate and adaptive immunity by activating antigen-presenting cells and lymphocytes, contributing to immune regulation and tissue repair [132,133].

The human peptide LL-37 is a well-studied example of an immune regulator. Its activity involves binding to bacterial components such as LPS, which prevents excessive macrophage stimulation and decreases TNF-α production. Furthermore, LL-37 disrupts the interaction between LPS and LBP (binding protein), reducing TLR4 activation and limiting the development of sepsis. The LL-37 peptide promotes the recruitment of immune cells by inducing chemokines such as IL-8 and MCP-1 and increasing the expression of chemokine receptors such as IL-8RB, CXCR-4, and CCR2 [134]. The peptide’s activity is dose-dependent, so at low concentrations it functions as an “immune sentinel,” while at higher levels it promotes cell migration. According to Fjell et al. [135], LL-37’s antimicrobial action is strengthened by its ability to directly penetrate bacterial membranes without the need for active endocytosis, whereas its immunomodulatory effects rely on its translocation into eukaryotic cells.

The peptides HV2 (RRVHVHVDPGVHVHVRR-NH2, synthetic) and PA-13-KIAKRIWKI LRRR, Synthetic- also exhibit immunomodulatory activity. Their mechanism involves neutralizing LPS from Gram-negative bacteria, thereby inhibiting TNF-α production in RAW264.7 macrophage cells. This action is essential for protecting the host against endotoxic shock, which can result from severe Gram-negative [136]. Another example of an immunomodulatory peptide is β-defensin 1 (MRTSYLLLFTLCLLLSEM ASGGNFLTGLGHRSDHYNCVSSGGQCLYSACPIFTKIQGTCYRGKAKCCK, human), which acts on macrophages infected with *Staphylococcus aureus* by activating the PI3K-AKT-NF-κB signaling pathway. Activation of this cascade leads to increased expression of proinflammatory cytokines such as TNF-α, IL-6, CXCL10, CD40, and RANTES. Inhibition of this pathway significantly reduces cytokine expression, underscoring its importance in the immune response. Moreover, eBD-1 stimulates the expression of phagocytosis-related proteins such as CD16 and paxillin, thereby promoting efficient pathogen clearance [54].

## 6. Clinical Trial of AMPs

Over 5000 antimicrobial peptides have been described, yet only 31 have reached preclinical trials, 38 are in phases I-III (Table 2), and 17 have been FDA-approved and are currently on the market (Table 3). This scenario is primarily attributable to the pharmacokinetic and regulatory challenges associated with the use of AMPs in vivo, such as selective toxicity, low bioavailability, proteolytic degradation, and a lack of standardization in establishing specific legislative guidelines [95,137,138]. Nevertheless, AMPs remain promising alternatives for treating antibiotic resistance because they offer rapid antimicrobial activity, fewer adverse reactions, and broad therapeutic applications. This has led to a growing search for peptides with desirable characteristics as well as for peptide-engineering techniques and delivery systems that optimize their pharmacological profile [95,137].

Peptides exhibit low bioavailability owing to their instability at acidic pH, enzymatic action, and poor intestinal mucosal absorption, which is why most AMPs are administered via injection [138]. However, this characteristic can be advantageous depending on the type of treatment, as demonstrated by surotomycin, an antibacterial peptide in phase III clinical trials (NCT01598311). This peptide exhibits minimal systemic absorption, making it ideal for treating *Clostridium difficile*-associated diarrhea and for achieving the required high drug concentrations in the colon. Additionally, surotomycin is a cyclic lipopeptide, ensuring greater stability and proteolytic resistance [65,157].

Most AMPs are administered via parenteral, representing almost 80% of peptide delivery, despite the potential benefits of oral delivery [138,158]. Among these routes, intravenous (IV) injections provide 100% bioavailability, reaching the maximum plasma concentration (Tmax) more rapidly than other methods. In contrast, subcutaneous (SC) administration exhibits slower absorption and delayed Tmax [158]. However, even when pharmacokinetic barriers are addressed, challenges such as rapid metabolism, protein binding, and conformational instability remain. To overcome these limitations, transdermal delivery has emerged as a promising non-invasive alternative, self-administration, avoiding gastrointestinal degradation and first-pass metabolism [138].

An important consideration is the potential cytotoxic effect of AMPs, which is associated with their low target specificity and biochemical properties such as hydrophobicity, amphipathicity, and charge. Excessive hydrophobicity increases lytic activity, whereas cationicity enhances the selectivity toward bacterial membranes [11,135,137]. Furthermore, studies have highlighted the importance of selecting appropriate in vivo and in vitro models, particularly for erythrocyte-based assays, since interspecies differences can affect the dose dependence of peptides [159]. Murepavadin is an example of an unsuccessful molecule that reached a phase III trial (NCT03582007) for the oral treatment of resistant *Pseudomonas aeruginosa* infections but exhibited dose-dependent nephrotoxicity [160,161].

To improve the success of clinical trials, certain strategies are employed, such as topical or inhalable administration, which minimize systemic exposure. Examples such as XOMA-629, Omiganan, and PL-5 are applied topically as sprays or creams and are in phase III clinical trials. Meanwhile, nanotechnology-based systems using metallic nanomaterials (e.g., gold and silver nanoparticles) or polymeric materials (e.g., chitosan and lipid-based formulations), particularly with techniques like oil-in-water nanoemulsions, liposomes, and micelles, can optimize AMP delivery, absorption, and half-life [162,163]. Nisin A, an oral AMP used in preclinical testing, demonstrated greater stability and antimicrobial activity against *Staphylococcus aureus* strains when formulated in chitosan/sodium alginate (CS/SA) microspheres [164].

Additionally, chemical modifications, such as substituting l-amino acids with their d-enantiomers, *N*-methylation, PEGylation, glycosylation, cyclization, and lipidation, can enhance AMP performance. These structural changes can improve solubility and stability in aqueous solutions, reduce renal clearance, extend antimicrobial function, and influence peptide activity through multiple mechanisms [165,166,167]. For example, oritavancin, a semisynthetic lipoglycopeptide antibiotic derived from vancomycin, is used to treat Gram-positive bacteria, including resistant strains. Its spectrum of activity and half-life are extended by its cyclic structure, lipophilic aromatic side chain, and amino acid d-enantiomers [167,168].

## 7. Computational Approaches for AMPs Discovery and Design

**Figure 3:**
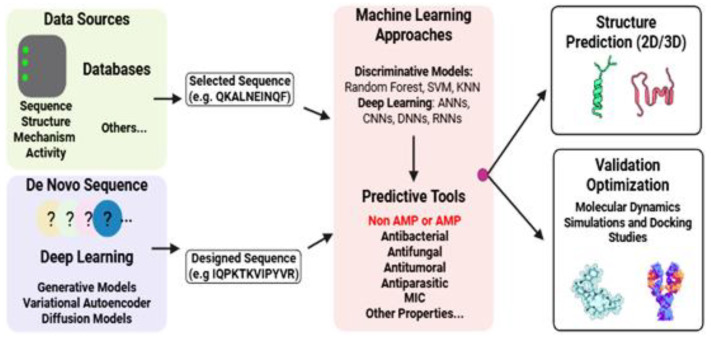
Two complementary strategies guide the discovery of antimicrobial peptides (AMPs): The identification of novel candidates from natural sources and the computationally guided design of synthetic AMPs. Protein images used in this figure were obtained from the freely accessible BioArt resource (https://bioart.niaid.nih.gov/).

Recent studies have shown notable advancements in computational methods for the discovery and optimization of AMPs. Existing research typically follows two complementary strategies: (1) bioinformatics-based mining of natural sequences to identify novel AMPs and (2) rational design of synthetic AMPs, either by modifying known templates through structure-based approaches or by employing de novo computational methods [169]. These strategies are summarized in Figure 3.

Quantitative structure–activity relationship (QSAR) modeling is one of the longest-standing computational approaches in AMP research. This method predicts biological activity on the basis of the molecular structure, operating under the principle that structurally similar compounds exhibit similar functions [170]. Traditional QSAR employs linear regression models, in which biological activity is calculated as the weighted sum of molecular descriptors. More advanced ML-based QSAR utilizes nonlinear algorithms, including random forest, artificial neural networks, K-nearest neighbor, and support vector machines, to capture complex structure–activity relationships through hierarchical feature interactions rather than simple weighted sums [171,172]. Overall, the previously cited ML strategies and other approaches (e.g., hidden Markov models, logistic regression, fuzzy K-nearest neighbor, discriminant analysis), have substantially enhanced AMP classification accuracy within a hierarchical multilevel description [173,174].

Discriminative models such as convolutional neural networks (CNNs), deep neural networks (DNNs), and recurrent neural networks (RNNs) are often integrated into pipelines for tasks such as AMP classification, property prediction, and bioactivity categorization [174,175]. Building on these advances, Brizuela et al. [176] identified five hierarchical levels of tool functionality: (1) distinguishing AMPs from non-AMPs, (2) categorizing inputs by bioactivity, (3) target classification and prediction, (4) predicting AMP properties, and (5) estimating minimum inhibitory concentrations (MICs).

With the emergence of new methods, researchers are employing approaches combining evolutionary algorithms, codon-based optimization, and AI to design AMPs with enhanced antimicrobial activity and reduced host toxicity [169]. For de novo AMP design, deep learning-based generative models, such as variational autoencoders (VAEs), diffusion models, and generative adversarial networks (GANs), have shown notable success when trained on large datasets of known AMPs. These models generate novel peptide sequences while optimizing desired properties [177].

Computational tools such as AlphaFold [178], RoseTTAFold [179,180] and PEP-FOLD [47] can be employed to predict AMP secondary and tertiary structures. Once structural models are available, molecular docking approaches, using platforms such as AutoDock [181], HADDOCK [182] and CABS-dock [183], allow the evaluation of potential interactions with target membranes or proteins. Subsequently, molecular dynamics simulations with packages such as GROMACS [184], CHARMM36 [185] or the coarse-grained MARTINI force field [186] provide insights into AMP stability, binding mechanisms, and dynamic behavior in biological environments. Table 4 shows the common tools for AMP analysis, supporting researchers in the identification of AMPs and the generation of novel peptide sequences.

Regardless of how the models are constructed, tool performance improves proportionally with database accuracy and proper structural organization [171,212]. Table 5 presents the widely used databases containing AMP data. These databases provide a comprehensive annotation on AMPs, including details such as amino acid sequence (composition and length), source organisms, biological activity, physicochemical properties (hydrophobicity and charge), secondary structures, mechanisms of action, and additional data [169].

## 8. AMPs and Their Use in the Food Industry

Antimicrobial peptides can be potentially used for food preservation, mainly because they are considered ecological, safe, and economically viable in comparison with chemical preservatives [41]. Moreover, plant- and animal-based foods can be enzymatically hydrolyzed to generate peptides with antimicrobial activity [42]. Research has highlighted the challenges in applying AMPs in food systems, such as the use of nanotechnology through nanoencapsulation, which can enhance stability, antimicrobial spectrum, and efficacy.

Microbial fermentation can generate BAPs from proteins of plant or animal origin. These peptides exhibit antimicrobial, antioxidant, antihypertensive, and anti-inflammatory properties, and show the potential for disease prevention and health promotion. Various microorganisms, including lactic acid bacteria, *Bacillus* spp., and fungi (*Aspergillus, Rhizopus, Saccharomyces*), can contribute to the release of these compounds during the fermentation of foods such as milk, meat, cereals, legumes, and seafood [223]. Bacteriocins are AMPs derived from probiotics and produced by lactic acid bacteria in traditional fermented foods. They have emerged as sustainable alternatives to antimicrobials and a means of food biopreservation [41,224]. These bacteriocins act by forming pores and interfering with vital cellular processes and are selective, which preserve the body’s beneficial microbiota [225].

The concept of “Clean Label” foods (foods produced with natural compounds) has gained prominence due to consumers’ demand for healthier diets. This growing demand has pressured the food industry to replace synthetic preservatives with sustainable alternatives [39]. In this context, the use of bacteriocins and protective cultures has emerged as a promising approach for food preservation. These active cultures release antimicrobial substances directly into food, thereby increasing microbiological safety through a comprehensive effect against harmful bacteria, and are compatible with food systems [226,227]. Despite these benefits, issues related to transparency, regulation, and social acceptance persist [228].

The combined use of probiotics and antimicrobials has been investigated as a novel approach to improve food safety and extend the shelf life of products. Probiotics can modulate microbiota, block pathogens, and simultaneously enhance the efficacy of AMPs. Synergistic approaches include the local production of antimicrobials through probiotic cultures and the strengthening of host immune defenses [229]. Analyses of toxicological risks, potential negative effects on beneficial microbiota, and mechanisms of bacterial resistance are important before these compounds can be used commercially [230]. Although these compounds have great potential as natural alternatives to artificial preservatives, information on their safety on a large scale is currently lacking. Obstacles include standardizing toxicological testing, investigating the effects on gut microbiota, and monitoring bacterial resistance [231]. From this perspective, innovation should be guided not only by technological efficiency but also by environmental sustainability, social justice, and public health protection [42].

## 9. AMPs and Their Use in Veterinary Medicine

The abuse and inappropriate use of antibiotics have caused AMR to become a major public health concern. One aspect of AMR has been discussed in Swann’s report published in 1969, which highlights the influence of AMR in animals and the latent concern that this may negatively affect humans [232].

In livestock animals, antibiotics are prophylactically used to promote growth. This approach employs sub-therapeutic doses, which are known to select resistant organisms [233]. The indiscriminate use of antibiotics to prevent and treat diseases generates selective pressure that could lead to the transfer of AMR microorganisms to humans as a result of the close relationship between the two. Wild animals may also aggravate this problem, since they could function as an ecological pool of resistant organisms; however, the transmission routes involving wild animals are difficult to untangle [234]. In this scenario, the application of AMPs in the veterinary field may substantially contribute to resolving the issue at hand, facilitating the treatment of pathologies and the modulation of the immune system [235].

Antimicrobial peptides can be directly applied as growth-promoting agents. A recombinantly expressed piscidin from *Epinephelus lanceolatus* was used to supplement chicken feed. Animals fed 1.5%, 3%, and 6% recombinant *E. lanceolatus* peptide showed better growth than those in the control groups, with an increase of approximately 15%, 20%, and 16% in weight gain, respectively [236]. Another study on piglets performed with recombinant bovine lactoferrampin–lactoferricin as a dietary supplement suggested that it may improve the digestive microbiome in weaned piglets [237]. Similarly, dietary supplementation of weaning pig feed with 60 mg/kg of the antimicrobial peptide P5 (KWKKLLKKPLLKKLLKKL-NH2, synthetic), improved nutrient digestibility and growth performance [238].

Antimicrobial peptides also have potential therapeutic applications. In a previous study, MPX (H-INWKGIAAMAKKLL-NH2), a peptide found in wasp venom, was found to counteract biofilm formation in vivo in a mouse model infected with *Actinobacillus pleuropneumoniae*, a highly infectious and relevant pathogen in the pig industry. This peptide destroyed the bacterial cell membrane and significantly reduced biofilm formation. MPX also prevented the death of mice infected with a lethal dose of *A. pleuropneumoniae* [239]. In a study conducted on chickens, the peptide HJH-3 (VNFKLLSHSLLVTLRSHL), derived from the bovine erythrocyte, showed good antibacterial activity against *Salmonella pullorum*, with low hemolysis and no cytotoxicity, and prevented death in chickens infected with a lethal dose of *S. pullorum* [240].

Furthermore, animals can be rich sources of AMPs, since these amino acid sequences are ubiquitous in the host defense system. Horses, for example, may be an interesting source of these substances, which encompass a wide range of peptides subdivided into several classes, including lysozymes, cathelicidins, defensins, and NK lysins [241]. Recently, a study integrating deep learning and bioinformatics attempted to describe new AMPs from the ruminant gastrointestinal microbiome, and the authors identified a peptide that can inhibit methicillin-resistant *Staphylococcus aureus* (MRSA). Fish are also a promising source of antimicrobial substances, since they are constantly exposed to pathogens [242].

Two recent reviews have focused on marine organisms and their application in aquaculture. García-Beltrán et al. [37] called attention to algae and cyanobacteria as sources of AMPs. Their study identified a wide range of activities, including antibacterial, antiparasitic, antifungal, antioxidant, and anti-inflammatory activities. Rodrigues et al. [38], on the other hand, focused on the AMPs derived from marine invertebrates. Their study identified approximately 350 peptides from 10 different invertebrate phyla, some of which showed broad-spectrum in vitro activities against pathogens, including fungi, Gram-positive bacteria, Gram-negative bacteria, and viruses. The only caveat described in their paper was the lack of in vivo studies reported in the literature, highlighting a research gap that should be addressed [38].

## 10. Conclusions

Considering the potential advantages of using BAPs as therapeutic agents, along with their market appeal, studies that refine the mode of action of these biomolecules have gained increasing importance, requiring both advanced research and clinical validation. The diversity of sources and methods to obtain BAPs makes them a rich and inexhaustible reservoir of possibilities for advancing human and veterinary health.

The application of omics techniques, which combine the results of bioinformatics analyses, has led to the identification of several peptide sequences with biological activities, enabling the generation of these peptides through chemical synthesis or gene expression. Several servers and databases are available to support research on biological activities and the design of peptide amino acid sequences. These bioinformatics tools, combined with AI, can predict the biological activity of a given amino acid sequence and suggest potential modifications to optimize the functional properties of AMPs.

## Figures and Tables

**Table 1:** Comparative summary of the main characteristics of peptide production methods.

Criterion	Chemical Synthesis	Enzymatic	Natural	Recombinant
Cost	High	Moderate	Variable	Low
Yield	Medium	Low	Very Low	High
Scalability	High	Moderate	Low	Very High
Technical Complexity	High	Low	Medium	High
Application Versatility	Very High	Moderate	Low	High
Level of Specificity	Very High	High	Moderate	High
Stability (Half-life)	High	Moderate	Variable	High

**Table 2:** Examples of biochemical characteristics of AMPs in clinical trials.

Peptide Name	Sequence	Structure	Source	Biological Activity	Target Site	Delivery Path	Molecular Weight	pI	Charge	Reference
Reltecimod, p2TA	ASPMLVAYDA	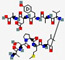	Synthetic	Antibacterial	Immune modulation	IV	1.0	3.8	−1	[58]
Ramoplanin, NTI-851	NNXXTXXXFXXXXGLAX	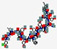	*Actinoplanes*	Antibacterial	Disrupt cell wall biosynthesis	Oral/Topical	1.5	5.52	0	[49]
XOMA-629	KLFRXQAKX	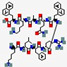	Synthetic	Antibacterial	Cell membrane	Topical cream	1.1	11.17	+3	[59]
Mycoprex	Not disclosed	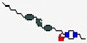	Insects	Antifungal	Cell membrane	IV	-	-	-	[60]
Demegal, D2A21	FAKKFAKKFKKFAKKFAKFAFAF	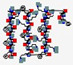	Synthetic	Antibacterial	Cell membrane	IV	2.7	10.9	+9	[61]
Glutoxim, NOV-002	DL-gGlu-DL-Cys-Gly-gGlu-DL-Cys-Gly.2Na+	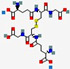	Synthetic	Antibacterial	Cell membrane	IV	0.68	3.8	−2	[62]
Omiganan, MBI-226	ILRWPWWPWRRK	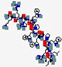	Synthetic	Antibacterial	Cell membrane	Topical	1.7	12.3	+4	[63]
Dusquetide SGX942	RIVPA	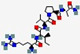	Synthetic	Antibacterial	Immune modulation	IV	0.55	9.75	+1	[64]
Surotomycin, MK-426	Not disclosed	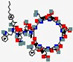	Actino bacteria	Antibacterial	Disrupt cytoplasmic membrane	Oral	-	-	-	[65]
Cefilavancin, TD-1792	Not disclosed	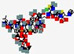	Synthetic	Antibacterial	Cell wall	IV	-	-	-	[66]
Peceleganan, PL-5	KWKSFLKTF KSA AKTVLHTALKAISS	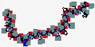	Synthetic	Antibacterial	Cell membrane	Topical spray	2.8	10.7	+6	[67]
Histatin	DXHEKRHHGYRRKFHEKHHSHREFPFYGDYGSNYLYDN	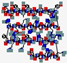	Human	Anti- candidal	Cell membrane/mitochondrion activity	Mouth wash	4.8	8.32	+1	[68]
PXL01	Not disclosed	-	Synthetic	Antibacterial	Inhibits inflammation	IV	-	-	-	[69]
Modimelanotide, AP-214	KKKKKKSYSMEHFRWGKPV	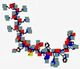	Synthetic	Antibacterial	Immune modulation	IV	2.3	10.47	+7	[70]
C16G2	TFFRLFNRSFTQALGKGGGKNLRIIRK GIHIIKKY	-	Synthetic	Antibacterial	Cell membrane	Mouth rinse	4	12.02	+9	[71]
Melantropin, CZEN-002	SYSMEHFRWGKPV	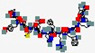	Synthetic	Anti-candidal	cAMP induction	Topical	1.6	8.33	+1	[72]
DPK-060	Not disclosed	*-*	Kininogen	Anti -infective	Membrane disruption	Topical	-	-	-	[73]
Ghrelin	GSSFLSPEHQRVQQRKESKKPPAKLQPR	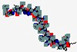	Synthetic	Anti-infective	Cell membrane	IV	3.2	11.07	+5	[74]
HXP124, Ppdef1	Not disclosed	-	Plant	Antifungal	Cell membrane	Topical	-	-	-	[75]
Inimex, IMX942	KSRIVPAIPVSLL	-	Synthetic	Anti-infective	Immune modulation	IV/	1.3	11	+2	[76]
Novexatin, NVXT	RRRRRRR	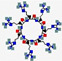	Synthetic	Antifungal	Cell wall	Topical	1.1	12.78	+7	[51]
OP-145	IGKEFKRIVERIKRFLRELVRPLR	-	Synthetic	Antibacterial	Lipid bilayer	Topical	3	11.72	+6	[77]
P113	AKRHHGYKRKFH	-	Human	Anti- infective	Cell membrane	Mouth wash	1.5	11.17	+5	[78]
PAC113	AKRHHGYKRKFH	-	Human	Antifungal	Target mitochondria	Oral rinse	1.5	11.17	+5	[79]
Bilacidin, PMX-30063	Not disclosed	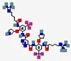	Synthetic	Antibacterial	Cell membrane	IV	1.	-	-	[80]
Sifuvirtide	SWETWEREIENYTRQIYRILEESQEQQDRN ERDLLE	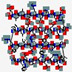	Synthetic	Anti-HIV	HIV fusion inhibitor	IV	4.6	4.33	−6	[81]
hLF1-11	GRRRRSVQWCA	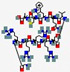	Humana	Antibacterial Antifungal	Inhibition of growth by iron scavenging	IV/ Topical	1.3	12	+4	[82]
LL-37	LLGDFFRKSKEKIGK EFKRIVQRIKDFLR NLVPRTES	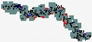	Human	Antibacterial	Membrane disruption	Topical/inhalation	4.4	10.61	+6	[83]
LTX-109	R-Tbt-R-NH-EtPh	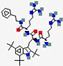	Synthetic	Antibacterial	Membrane disruption and cell lysis.	Topical (nasal)	0.78	-	-	[84]
Lotilibcin, WAP-8294A2	cyclo[D-Asn-D-Trp-D-Orn -N(Me)Val-ObAla(3R-isohexyl) -Ser-D-Asn- Ser-Gly- D- N(Me)Phe-Leu-D-Orn-Glu]	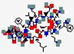	Lysobacter species	Antibacterial	Membrane disruption	IV	1.5	-	-	[85]
Opebacan	Not disclosed	-	Recombinant BPI fragment	Antibacterial Antiviral	Permeability increasing protein	IV	-	-	-	[86]
Friulimicin B	Not disclosed	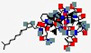	*Actinoplanes friuliensis*	Antibacterial	Cell membrane	IV	1.3	-	-	[87]
IDR-1	KSRIVPAIPVSLL-NH2	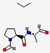	Synthetic	Anti-infective	Reduction of pro-inflammatory cytokines	IV/ Topical	1.3	11	+2	[88]
NVB302	Not disclosed	-	Synthetic	Antifungal	Lipid bilayer	IV	-	-	-	[89]
Plectasin, NZ2114	GFGCNGPWDEDDM QCHNHCKSIKGYK GGYCAK GGFVCKCY	-	*Pseudoplectania nigrella*	Antibacterial	Lipid bilayer	IV/ Topical	4.4	7.77	+1	[90]
Vasoactive intestinal peptide	HSDAVFTDNYTRLRKQMAVKKYLNSILN	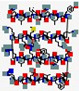	Human	Antibacterial	G-protein-coupled receptors	IV/Inhalation	3.3	9.82	+3	[91]

(-) no information. Molecular weight is represented in kDa. All information was collected and reviewed in the DRAMP database (http://dramp.cpu-bioinfor.org/), Chinese Clinical Trial Registry [ChiCTR] (https://www.chictr.org.cn/indexEN.html), European Union Clinical Trials Register (https://www.clinicaltrialsregister.eu/ctr-search/search), ClinicalTrials.gov (https://www.clinicaltrials.gov/) (accessed 28 August 2025).

**Table 3:** AMPs approved for FDA and in market.

**Peptide Name**	Sequence	Structure	Source	Biological Activity	Medical Use	Target Site	Delivery Path	Company	**Reference**
Bacitracin	Leu-D-Glu Ile-Lys-D-Orn-Ile-D-Phe-His-D-Asp-Asn	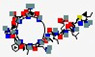	*Bacillus licheniformis*	Antibacterial	Prevent pneumonia and empyema in infants, skin and eye infections.	Cell wall	Topical	Various companies	[139]
Dalbavacin Xydalba	Not available	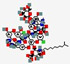	Semi synthetic	Antibacterial	Acute bacterial skin infections, Osteomyelitis and septic arthritis	Cell wall	IV	DALVANCE^®^	[140]
Daptomycin Cubicin	Decanoyl-WNTGO DaDGsED	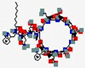	*Streptomyces roseosporus*	Antibacterial	Complicated skin infections and bloodstream infections (bacteremia)	Cell membrane	IV	CUBICIN^®^	[141]
Enfuvirtide Fuzeon T20	YTSLIHSLEESQNQQEKNEQELLELDKWASLWNWF	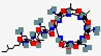	Synthetic	Anti-HIV	Human Immunodeficiency Virus (HIV)	Fusion protein gp41	SC	FUZEON^®^	[142]
Gramicidin D	VGALAVVVWLW LWLW-ethanolamine	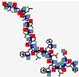	*Bacillus brevis*	Antibacterial	Skin lesions and eye infections	Cell membrane	Topical	Various companies	[143]
Gramicidin S	cyclo[Leu-D -Phe -Pro-Val-Orn-Leu-D-Phe-Pro-Val-Orn]	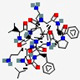	*Bacillus brevis*	Antibacterial	Spermicide against bacteria and fungi; genital ulcers	Cell membrane	Topical	Various companies	[144]
Obiltoxaximab	Not available	Not available	monoclonal antibody	Antibacterial	Inhalational anthrax	Antitoxin	IV	ANTHIM^®^	[145]
Oritavancin	Not available	Not available	Semi synthetic	Antibacterial	Acute bacterial skin	Cell wall	IV	KIMYRSA™ ORBACTIV^®^	[146]
Palivizumab	Not available	Not available	monoclonal antibody	Antiviral	Prevent serious lung infections caused by respiratory syncytial virus-RSV	Blocking viral replication	IM	SYNAGIS^®^	[147]
Polymyxin B	Not available	Not available	*Bacillus polymyxa*	Antibacterial	Infections of the urinary tract, meninges, and blood stream	Cell membrane	IV	Various companies	[148]
Polymyxin E Colistin	6-mh-DabTDab [Î^3^DablLDabDabT]	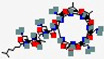	*Bacillus polymyxa*	Antibacterial	Acute or chronic infections due to Gram-negative bacilli	Cell membrane	Oral	Various companies	[149]
Raxibacumab	Not available	Not available	monoclonal antibody	Antibacterial	Inhalational anthrax	Antitoxin	IV	RAXIBACUMAB ^®^	[11]
Telavancin TD-6424	Not available	Not available -	Semi synthetic	Antibacterial	Osteomyelitis and bacterial infections	Cell membrane	IV	VIBATIV^®^	[150]
Tyrothricin	Not available	Not available -	*Brevibacillus parabrevis*	Antibacterial Antifungal	Infected skin and infected oropharyngeal mucous membranes	Cell membrane	Topical/ Oral	Various companies	[151,152]
Thymalfasin	SDAAVDTSSEITTKDLKEKKEVVEEAE N	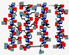	Synthetic	Antiviral	Hepatitis B and C.	Immuno modulator	IV	ZADAXIN^®^	[153]
Vancomycin	Not available	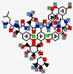	*Amycolatopsis orientalis*	Antibacterial	Septicemia, infective endocarditis, skin, bone and lower respiratory tract infections.	Cell wall	IV/Oral	Various companies	[154]

Intravenous injections (IV), Subcutaneous injections (SC) Intramuscular injections (IM). All information was collected and reviewed in the DRAMP database (http://dramp.cpu-bioinfor.org/) [16], Drugbank (https://go.drugbank.com/) [155] and Pubchem (https://pubchem.ncbi.nlm.nih.gov/) [156] (accessed 3 August 2025).

**Table 4:** AMPs tools for analysis.

Database Name	Functionality	Additional Information	URL	Reference
Prediction of whether it is an AMP and its potential activity.				
ACEP	Identification of AMPs.	The classification method is based on deep learning (DL).	https://github.com/Fuhaoyi/ACEP (accessed 30 October 2025)	[187]
ADAPTABLE	Designing novel peptides, predicting their activities, and identifying functional motifs.	Webserver and data-miner of antimicrobial peptides.	http://gec.upicardie.fr/adaptable/ (accessed 30 October 2025)	[188]
AFP-MFL	A novel deep learning model that can predict antifungal peptides.	It only needs the peptide sequence to run.	https://inner.wei-group.net/AFPMFL/#/ (accessed 30 October 2025)	[189]
AMP- Scanner	A web server tool for predicting if it is an AMP based on amino acid sequence.	Only includes bacteria targets.	https://www.dveltri.com/ascan/ (accessed 30 October 2025)	[190]
AMPA	A web server tool for identifying active regions in antimicrobial proteins.	The algorithm uses an antimicrobial propensity scale to generate an antimicrobial profile.	https://tcoffee.crg.eu/apps/ampa/do (accessed 30 October 2025)	[191,192]
AMPDeep	Deep learning approach to predict hemolytic activity of AMPs.	It was built on Python.	https://github.com/milad73s/AMPDeep (accessed 30 October 2025)	[193]
amPEPpy	A python application for predicting antimicrobial peptide sequences.	The classification method is based on random forest.	https://github.com/tlawrence3/amPEPpy (accessed 30 October 2025)	[194]
AmpGram	A web server tool for identification of AMPs.	The classification method is based on random forest and n-gram analysis.	http://biongram.biotech.uni.wroc.pl/AmpGram/ (accessed 30 October 2025)	[195]
AxPEP	It is a collection of sequence-based machine learning methods for AMPs prediction.	The classification method is based on random forest.	https://app.cbbio.online/ampep/home (accessed 30 October 2025)	[196]
CS-AMPPred	An SVM-based (support vector machines) tool to predict antimicrobial activity in cysteine-knotted proteins.	Were based on 310 AMPs and 310 non-antimicrobial peptide sequences.	https://sourceforge.net/projects/csamppred/ (accessed 30 October 2025)	[197]
Cysmotif Searcher	A Perl package for revealing peptide sequences possessing cysteine motifs.	Cysteine motifs are common to various families of AMPs and other cysteine-rich peptides.	https://github.com/fallandar/cysmotifsearcher (accessed 30 October 2025)	[198]
deepAMP	A tool for predicting protein AMPylation sites from binary profile representation.	The classification method is based on a convolutional neural network.	https://github.com/MehediAzim/DeepAmp (accessed 30 October 2025)	[199]
iAMPCN	DL approach to identifying antimicrobial peptides and their functional activities.	It was built on Python.	https://github.com/joy50706/iAMPCN (accessed 30 October 2025)	[200]
MLBP	Multi-label DL approach to identifying multi-functional bioactive peptide functions.	It was built on Python.	https://github.com/tangwending/MLBP (accessed 30 October 2025)	[201]
sAMPpred-GAT	A web tool for identification of AMPs.	The program uses graphs constructed based on predicted peptide structures.	http://bliulab.net/sAMPpred-GAT/server (accessed 30 October 2025)	[202]
Design of Peptides				
AntiBP 3.0	A web-tool for predicting, scanning and designing AMPs.	Based on machine learning techniques.	https://webs.iiitd.edu.in/raghava/antibp3/ (accessed 30 October 2025)	[203]
Joker	An algorithm to design antimicrobial peptides.	It was developed based on amino acid motifs.	https://github.com/williamfp7/Joker (accessed 30 October 2025)	[204]
ModlAMP	A Python package for working with any sequence of natural amino acids.	It includes the following modules: sequence generation, sequence library analysis and description calculation.	https://modlamp.org/ (accessed 30 October 2025)	[205]
Prediction of Structure				
AlphaFold	To predict a protein/peptide’s 3D structure based on amino acid sequence.	AI system developed by Google DeepMind.	https://colab.research.google.com/github/sokrypton/ColabFold/blob/main/AlphaFold2.ipynb (accessed 30 October 2025)	[206,207]
iTASSER	A web tool for predicting structure of proteins.	It also provides a structure-based function annotation.	https://zhanggroup.org/I-TASSER/ (accessed 30 October 2025)	[208]
PEP2D	A web tool for predicting secondary structure of peptides.	The model was trained and tested based on 3100 peptide structures.	https://webs.iiitd.edu.in/raghava/pep2d/ (accessed 30 October 2025)	[209]
PEP-FOLD 4	A de novo approach to predict peptide structure from amino acid sequences.	The peptides should have 5 to 50 amino acids.	https://mobyle2.rpbs.univ-paris-diderot.fr/cgi-bin/portal.py#forms::PEP-FOLD4 (accessed 30 October 2025)	[209]
RoseTTAFold	A Python-based AI tool for protein/peptide structure prediction.	The structure prediction method is based on DL.	https://github.com/RosettaCommons/RoseTTAFold?tab=readme-ov-file (accessed 30 October 2025)	[179]
SWISS-MODEL	A web tool for protein structure prediction and modeling.	It supports interactive modeling for both simple and complex needs.	wissmodel.expasy.org (accessed 30 October 2025)	[210]
Molecular Docking and Molecular Modeling				
AutoDock	A tool to predict how small molecules bind to a receptor of know 3D structure.	It has two options: AutoDock-GPU and AutoDock Vina.	https://autodock.scripps.edu/ (accessed 30 October 2025)	[181]
CABS-dock	A web server tool for flexible protein-peptide docking.	It requires the sequence of a protein receptor and a peptide sequence.	https://biocomp.chem.uw.edu.pl/CABSdock (accessed 30 October 2025)	[183]
CHARMM36	Broad-scope molecular simulation software for complex environments.	There is no cost to academic students.	https://academiccharmm.org/ (accessed 30 October 2025)	[185]
GROMACS	Powerful open-source suite for molecular dynamics simulation and analysis.	Broad spectrum of calculation types, preparation and analysis tools.	https://manual.gromacs.org/archive/4.6.7/online/speptide.html (accessed 30 October 2025)	[184]
HADDOCK	A web platform for biomolecular docking simulations.	A data-driven docking approach guided by ambiguous interaction restraints (AIRs).	https://rascar.science.uu.nl/haddock2.4 (accessed 30 October 2025)	[97]
MARTINI	Coarse-grained force field for biomolecular simulations, parameterized using experimental data and atomistic simulations.	Based on a four-to-one mapping scheme.	https://cgmartini.nl/ (accessed 30 October 2025)	[186]
NAMD	A highly scalable software for parallel molecular dynamics simulations of large biomolecules.	It uses the program VHD for simulation setup and trajectory analysis.	https://www.ks.uiuc.edu/Research/namd/ (accessed 30 October 2025)	[211]
VMD	A program to display, animate and analyze large biomolecules systems.	It has 3-D graphics.	https://www.ks.uiuc.edu/Research/vmd/ (accessed 30 October 2025)	[5]

**Table 5:** AMPs databases.

Database Name	Type of data	Additional Information	URL	Reference
ADAM	Comprehensive AMPs database.	It contains tools for searching and predicting whether peptides are antimicrobial.	http://bioinformatics.cs.ntou.edu.tw/adam/index.html (accessed 30 October 2025)	[213]
AMPDb	Extensively curated AMPs.	It assimilates information from various resources: NCBI, EMBL, UniProt, RCSB-PDB and PubMed.	https://bblserver.org.in/ampdb/ (accessed 30 October 2025)	[17]
APD	Manually curated AMPs.	It contains 5099 peptides	https://aps.unmc.edu/ (accessed 30 October 2025)	[8]
CAMP	Conserved sequence signatures represented by 45 families.	It includes sequence, protein definition, accession numbers, activity, source organism, target organisms and protein family descriptions	https://classamp.bicnirrh.res.in/ (accessed 30 October 2025)	[13]
CARD	Antibiotics, resistance genes, their products and associated phenotypes.	It includes tools as BLAST and can predict resistome based on homology and SNP models.	https://card.mcmaster.ca/ (accessed 30 October 2025)	[214]
DADP	Manually curated database of AMPs and defense peptides.	Described minimum-inhibiting concentration (MIC) against at least one microorganism.	https://webs.iiitd.edu.in/raghava/satpdb/catalogs/dadp/ (accessed 30 October 2025)	[215]
dbAMP	Manually curated AMPs.	It includes tools that can discover novel AMPs or relate functional activities by predictive models.	http://awi.cuhk.edu.cn/dbAMP (accessed 30 October 2025)	[216]
DBAASP	Structure–activity of peptides.	It includes a tool that predicts whether the amino acid sequence has antimicrobial potential.	https://dbaasp.org/home (accessed 30 October 2025)	[12]
DRAMP	Manually curated AMPs.	It includes the annotations: sequences, structures, activities, physicochemical, patent, and clinical	http://dramp.cpu-bioinfor.org/ (accessed 30 October 2025)	[16]
Hemolytik	Manually curated peptides, including AMPs.	It contains experimentally validated hemolytic and non-hemolytic peptides.	http://crdd.osdd.net/raghava/hemolytik/ (accessed 30 October 2025)	[217]
InverPep	Specialized database of AMPs from invertebrates.	It includes extensive annotations: name, sequence, length, secondary structure, molar mass, charge, isoelectric point, hydrophobicity, etc.	https://ciencias.medellin.unal.edu.co/gruposdeinvestigacion/prospeccionydisenobiomoleculas/InverPep/public/home_en (accessed 30 October 2025)	[218]
MEGARes	Hand-curated resistance genes for antimicrobial drugs, biocides and metals.	Sources include Antibacterial Biocide and Metal Resistance Genes (BacMet), CARD, ResFinder, PointFinder, AMRFinderPlus.	https://www.meglab.org/megares/ (accessed 30 October 2025)	[219]
PlantPepDB	Information about plant peptides	Users can get all information about different functional, physicochemical and structural properties of a single peptide, in a single entry.	http://14.139.61.8/PlantPepDB/pages/simplesearch.php (accessed 30 October 2025)	[18]
Peptipedia	Tool for peptide sequence	Uses 76 databases and reports on activity and sequence	https://app.peptipedia.cl/ (accessed 30 October 2025)	[15]
PepTherDia	Manually curated database of approved peptide drugs and diagnostic agents.	Annotations: structural, physicochemical and pharmacokinetic properties, indications, routes of administration, production methodologies, marketing authorization and origin of their design.	https://peptherdia.herokuapp.com/ (accessed 30 October 2025)	[220]
SATPdb	Structurally annotated therapeutic peptides.	It is possible to search for all the peptides containing a common segment (motif) or well-known motifs.	https://webs.iiitd.edu.in/raghava/satpdb/sub_search.php (accessed 30 October 2025)	[14]
ThPDB	Approved and approved/investigational therapeutic peptides.	It includes sequence, indication, mechanism of action, pharmacodynamics, toxicity, metabolism, absorption, half-life, patent information, interaction with other drugs, targets, others.	http://crdd.osdd.net/raghava/thpdb/ (accessed 30 October 2025)	[221]
YADAMP	Structurally annotated peptides classified into 10 categories.	Include anticancer, antiviral, antiparasitic, antibacterial, drug delivery and toxic peptides.	https://webs.iiitd.edu.in/raghava/satpdb/index.html (accessed 30 October 2025)	[222]

All information was collected and reviewed on 5 October.

## Data Availability

No new data were created or analyzed in this study. Data sharing is not applicable to this article.

## Abbreviations

BAP	Bioactive Peptide
AMP	Antimicrobial Peptide
APD3	Antimicrobial Peptide Database 3
AMR	Antibiotic Resistance
WHO	World Health Organization
MERS	Middle East respiratory syndrome
SARS	Severe acute respiratory syndrome
AVP	Antiviral peptides
AFP	Antifungal peptides
SPPS	Solid-phase Peptide Synthesis
HBTU	O-benzotriazole-N,N,N′,N′-tetramethyl-uronium-hexafluoro-phosphate
HATU	O-(7-azabenzotriazol-1-yl)-N,N,N′,N′-tetramethyluronium hexafluorophosphate
DIC	N,N′-diisopropylcarbodiimide
CCK	Cyclic Cystine Knot
DMF	Dimethylformamide
UAE	Ultrasound-assisted extraction
PEF	Pulsed electric fields
HHP	High hydrostatic pressure
AI	Artificial Intelligence
ML	Machine Learning
GST	Glutathione S-transferase
MBP	Maltose-binding protein
GRAS	Generally recognized as safe
PG	Phosphatidylglycerol
CL	Cardiolipin
PS	Phosphatidylserine
LPS	Lipopolysaccharides
HSPG	Heparan sulfate proteoglycan
HCMV	Human cytomegaloviruses
MCMV	Murine cytomegaloviruses
DHFR	Dihydrofolate reductase
TCA	Tricarboxylic acid
SQR	Succinate-coenzyme Q reductase
ROS	Reactive oxygen species
HSV-1	Simplex virus type 1
HDPs	Host defense peptides
LBP	Binding protein
FDA	Food and Drug Administration
IV	Intravenous injections
IM	Intramuscular injections
SC	Subcutaneous injections
CS/SA	Chitosan/sodium alginate
QSAR	Quantitative Structure–Activity Relationship
CNNs	Convolutional Neural Networks
DNNs	Deep Neural Networks
RNNs	Recurrent Neural Networks
VAEs	Variational Autoencoders
GANs	Generative Adversarial Networks
MRSA	Methicillin-resistant *Staphylococcus aureus*
DL	Deep learning
SVM	Support vector machines
AIR	Ambiguous interaction restraints (AIRs).

## References

[1] Bizzotto E., Zampieri G., Treu L., Filannino P., Di Cagno R., Campanaro S. (2024). Classification of bioactive peptides: A systematic benchmark of models and encodings. Comput. Struct. Biotechnol. J..

[2] Lau J.L., Dunn M.K. (2018). Therapeutic peptides: Historical perspectives, current development trends, and future directions. Bioorganic Med. Chem..

[3] Kanaujia K.A., Wagh S., Pandey G., Phatale V., Khairnar P., Kolipaka T., Rajinikanth P., Saraf S.A., Srivastava S., Kumar S. (2025). Harnessing marine antimicrobial peptides for novel therapeutics: A deep dive into ocean-derived bioactives. Int. J. Biol. Macromol..

[4] Habib H.M., Ismail R., Agami M., El-Yazbi A.F. (2025). Exploring the impact of bioactive peptides from fermented Milk proteins: A review with emphasis on health implications and artificial intelligence integration. Food Chem..

[5] Sánchez A., Vázquez A. (2017). Bioactive peptides: A review. Food Qual. Saf..

[6] Van Epps H.L. (2006). René Dubos: Unearthing antibiotics. J. Exp. Med..

[7] Boparai J.K., Sharma P.K. (2019). Mini Review on Antimicrobial Peptides, Sources, Mechanism and Recent Applications. Protein Pept. Lett..

[8] Wang G., Li X., Wang Z. (2016). APD3: The antimicrobial peptide database as a tool for research and education. Nucleic Acids Res..

[9] Brogden K.A. (2005). Antimicrobial peptides: Pore formers or metabolic inhibitors in bacteria?. Nat. Rev. Microbiol..

[10] Roudi R., Syn N.L., Roudbary M. (2017). Antimicrobial Peptides as Biologic and Immunotherapeutic Agents against Cancer: A Comprehensive Overview. Front. Immunol..

[11] Lei J., Sun L., Huang S., Zhu C., Li P., He J., Mackey V., Coy D.H., He Q. (2019). The antimicrobial peptides and their potential clinical applications. Am. J. Transl. Res..

[12] Pirtskhalava M., Amstrong A., Grigolava M., Chubinidze M., Alimbarashvili E., Vishnepolsky B., Gabrielian A., Rosenthal A., Hurt D., Tartakovsky M. (2021). DBAASP v3: Database of antimicrobial/cytotoxic activity and structure of peptides as a resource for development of new therapeutics. Nucleic Acids Res..

[13] Gawde U., Chakraborty S., Waghu F.H., Barai R.S., Khanderkar A., Indraguru R., Shirsat T., Idicula-Thomas S. (2023). CAMPR4: A database of natural and synthetic antimicrobial peptides. Nucleic Acids Res..

[14] Singh S., Chaudhary K., Dhanda S.K., Bhalla S., Usmani S.S., Gautam A., Tuknait A., Agrawal P., Mathur D., Raghava G.P. (2016). SATPdb: A database of structurally annotated therapeutic peptides. Nucleic Acids Res..

[15] Quiroz C., Saavedra Y.B., Armijo-Galdames B., Amado-Hinojosa J., Olivera-Nappa Á., Sanchez-Daza A., Medina-Ortiz D. (2021). Peptipedia: A user-friendly web application and a comprehensive database for peptide research supported by Machine Learning approach. Database.

[16] Ma T., Liu Y., Yu B., Sun X., Yao H., Hao C., Li J., Nawaz M., Jiang X., Lao X. (2025). DRAMP 4.0: An open-access data repository dedicated to the clinical translation of antimicrobial peptides. Nucleic Acids Res..

[17] Mondal R.K., Sen D., Arya A., Samanta S.K. (2023). Developing anti-microbial peptide database version 1 to provide comprehensive and exhaustive resource of manually curated AMPs. Sci. Rep..

[18] Das D., Jaiswal M., Khan F.N., Ahamad S., Kumar S. (2020). PlantPepDB: A manually curated plant peptide database. Sci. Rep..

[19] O’Neill J.C. (2016). Tackling Drug-Resistant Infections Globally: Final Report and Recommendations. Review on Antimicrobial Resistance. https://amr-review.org/sites/default/files/160525_Final%20paper_with%20cover.pdf.

[20] World Health Organization (2024). Pathogens Prioritization A.

[21] Poudel A.N., Zhu S., Cooper N., Little P., Tarrant C., Hickman M., Yao G. (2023). The economic burden of antibiotic resistance: A systematic review and meta-analysis. PLoS ONE.

[22] Al-Madboly L.A., Aboulmagd A., El-Salam M.A., Kushkevych I., El-Morsi R.M. (2024). Microbial enzymes as powerful natural anti-biofilm candidates. Microb. Cell Factories.

[23] Yang A., Bai Y., Zhang Y., Xiao R., Zhang H., Chen F., Zeng W. (2025). Detection and Treatment with Peptide Power: A New Weapon Against Bacterial Biofilms. ACS Biomater. Sci. Eng..

[24] World Health Organization (2025). Disease Outbreak News; Ebola Virus Disease in the Democratic Republic of the Congo. https://www.who.int/emergencies/disease-outbreak-news/item/2025-DON580.

[25] Qureshi A. (2025). A review on current status of antiviral peptides. Discov. Viruses.

[26] Puumala E., Fallah S., Robbins N., Cowen L.E. (2024). Advancements and challenges in antifungal therapeutic development. Clin. Microbiol. Rev..

[27] Sharma A., Lee H.-J. (2025). Antimicrobial Activity of Probiotic Bacteria Isolated from Plants: A Review. Foods.

[28] Rivera-Fernández N., Anacleto-Santos J., Casarrubias-Tabarez B., López-Pérez T.d.J., Rojas-Lemus M., López-Valdez N., Fortoul T.I. (2022). Bioactive Peptides against Human Apicomplexan Parasites. Antibiotics.

[29] Huan Y., Kong Q., Mou H., Yi H. (2020). Antimicrobial Peptides: Classification, Design, Application and Research Progress in Multiple Fields. Front. Microbiol..

[30] Proaño-Bolaños C., Morán-Marcillo G., Monteros-Silva N.E.d.L., Bermúdez-Puga S., Salazar M.A., Blasco-Zúñiga A., Cuesta S., Molina C., Espinosa F., Meneses L. (2024). Bioactivity of synthetic peptides from Ecuadorian frog skin secretions against *Leishmania mexicana*, *Plasmodium falciparum*, and *Trypanosoma cruzi*. Microbiol. Spectr..

[31] Slezina M.P., Odintsova T.I. (2023). Plant Antimicrobial Peptides: Insights into Structure-Function Relationships for Practical Applications. Curr. Issues Mol. Biol..

[32] Shirsat H., Datt M., Kale A., Mishra M. (2025). Plant Defense Peptides: Exploring the Structure–Function Correlation for Potential Applications in Drug Design and Therapeutics. ACS Omega.

[33] Meng T., Wen J., Liu H., Guo Y., Tong A., Chu Y., Du B., He X., Zhao C. (2025). Algal proteins and bioactive peptides: Sustainable nutrition for human health. Int. J. Biol. Macromol..

[34] García-Encinas J.P., Ruiz-Cruz S., Juárez J., Ornelas-Paz J.d.J., Del Toro-Sánchez C.L., Márquez-Ríos E. (2025). Proteins from Microalgae: Nutritional, Functional and Bioactive Properties. Foods.

[35] Bosso A., Di Nardo I., Culurciello R., Palumbo I., Gaglione R., Zannella C., Pinto G., Siciliano A., Carraturo F., Amoresano A. (2025). KNR50: A moonlighting bioactive peptide hidden in the C-terminus of bovine casein αS2 with powerful antimicrobial, antibiofilm, antiviral and immunomodulatory activities. Int. J. Biol. Macromol..

[36] Kandasamy S.K., Larson R.G. (2004). Binding and insertion of α-helical anti-microbial peptides in POPC bilayers studied by molecular dynamics simulations. Chem. Phys. Lipids.

[37] García-Beltrán J.M., Arizcun M., Chaves-Pozo E. (2023). Antimicrobial Peptides from Photosynthetic Marine Organisms with Potential Application in Aquaculture. Mar. Drugs.

[38] Rodrigues T., Guardiola F.A., Almeida D., Antunes A. (2025). Aquatic Invertebrate Antimicrobial Peptides in the Fight Against Aquaculture Pathogens. Microorganisms.

[39] Chauhan K., Rao A. (2024). Clean-label alternatives for food preservation: An emerging trend. Heliyon.

[40] Hao Y., Teng D., Mao R., Yang N., Wang J. (2023). Site Mutation Improves the Expression and Antimicrobial Properties of Fungal Defense. Antibiotics.

[41] Bahrami S., Andishmand H., Pilevar Z., Hashempour-Baltork F., Torbati M., Dadgarnejad M., Rastegar H., Mohammadi S.A., Azadmard-Damirchi S. (2024). Innovative perspectives on bacteriocins: Advances in classification, synthesis, mode of action, and food industry applications. J. Appl. Microbiol..

[42] Fabián J.C.P., Contreras A.K.Á., Bonifacio I.N., Robles M.F.H., Quiñones C.R.V., Ramírez E.I.Q., Salinas C.V. (2025). Toward safer and sustainable food preservation: A comprehensive review of bacteriocins in the food industry. Biosci. Rep..

[43] Esposito D., Chatterjee D.K. (2006). Enhancement of soluble protein expression through the use of fusion tags. Curr. Opin. Biotechnol..

[44] Bernardeau M., Vernoux J.P., Henri-Dubernet S., Guéguen M. (2008). Safety assessment of dairy microorganisms: The Lactobacillus genus. Int. J. Food Microbiol..

[45] Sorokulova I. (2013). Modern Status and Perspectives of Bacillus Bacteria as Probiotics. J. Probiotics Health.

[46] Sutcliffe R., Doherty C.P.A., Morgan H.P., Dunne N.J., McCarthy H.O. (2025). Strategies for the design of biomimetic cell-penetrating peptides using AI-driven in silico tools for drug delivery. Biomater. Adv..

[47] Shen Y., Maupetit J., Derreumaux P., Tufféry P. (2014). Improved PEP-FOLD Approach for Peptide and Miniprotein Structure Prediction. J. Chem. Theory Comput..

[48] Ludtke S.J., He K., Heller W.T., Harroun T.A., Yang L., Huang H.W. (1996). Membrane Pores Induced by Magainin. Biochemistry.

[49] Sugimoto A., Maeda A., Itto K., Arimoto H. (2017). Deciphering the mode of action of cell wall-inhibiting antibiotics using metabolic labeling of growing peptidoglycan in Streptococcus pyogenes. Sci. Rep..

[50] Cursino L., Smajs D., Smarda J., Nardi R., Nicoli J., Chartone-Souza E., Nascimento A. (2006). Exoproducts of the Escherichia coli strain H22 inhibiting some enteric pathogens both in vitro and in vivo. J. Appl. Microbiol..

[51] Mercer D.K., Robertson J.C., Miller L., Stewart C.S., O’Neil D.A. (2020). NP213 (Novexatin®): A unique therapy candidate for onychomycosis with a differentiated safety and efficacy profile. Med. Mycol..

[52] Lewies A., Wentzel J.F., Garmi J., Du Plessis L.H. (2015). The Potential Use of Natural and Structural Analogues of Antimicrobial Peptides in the Fight against Neglected Tropical Diseases. Molecules.

[53] Grover A., Singh S., Sindhu S., Lath A., Kumar S. (2025). Advances in cyclotide research: Bioactivity to cyclotide-based therapeutics. Mol. Divers..

[54] Pei L., Hou Y., Feng Y., Li F., Su H., Zhang Y., Song Y., Liu K., Cao G. (2023). Equine β-defensin 1 regulates cytokine expression and phagocytosis in S. aureus-infected mouse monocyte macrophages via the Paxillin-FAK-PI3K pathway. Int. Immunopharmacol..

[55] Lundbæk J.A., Collingwood S.A., Ingólfsson H.I., Kapoor R., Andersen O.S. (2010). Lipid bilayer regulation of membrane protein function: Gramicidin channels as molecular force probes. J. R. Soc. Interface.

[56] Bahar A., Ren D. (2013). Antimicrobial Peptides. Pharmaceuticals.

[57] Pasupuleti M., Schmidtchen A., Malmsten M. (2012). Antimicrobial peptides: Key components of the innate immune system. Crit. Rev. Biotechnol..

[58] Bulger E.M., Maier R.V., Sperry J., Joshi M., Henry S., Moore F.A., Moldawer L.L., Demetriades D., Talving P., Schreiber M. (2014). A Novel Drug for Treatment of Necrotizing Soft-Tissue Infections. JAMA Surg..

[59] Mba I.E., Nweze E.I. (2022). Antimicrobial Peptides Therapy: An Emerging Alternative for Treating Drug-Resistant Bacteria. Yale J. Biol. Med..

[60] Jadi P.K., Sharma P., Bhogapurapu B., Roy S. (2021). Alternative Therapeutic Interventions: Antimicrobial Peptides and Small Molecules to Treat Microbial Keratitis. Front. Chem..

[61] Shriwastav S., Kaur N., Hassan M., Ahmed Mohammed S., Chauhan S., Mittal D., Shahbaz A., Bibi A. (2025). Antimicrobial peptides: A promising frontier to combat antibiotic resistant pathogens. Ann. Med. Surg..

[62] Kirkpatrick D.L., Powis G. (2017). Clinically Evaluated Cancer Drugs Inhibiting Redox Signaling. Antioxid. Redox Signal..

[63] You Y., Liu H., Zhu Y., Zheng H. (2023). Rational design of stapled antimicrobial peptides. Amino Acids.

[64] North J.R., Takenaka S., Rozek A., Kielczewska A., Opal S., Morici L.A., Finlay B., Schaber C.J., Straube R., Donini O. (2016). A novel approach for emerging and antibiotic resistant infections: Innate defense regulators as an agnostic therapy. J. Biotechnol..

[65] Chandorkar G., Zhan Q., Donovan J., Rege S., Patino H. (2017). Pharmacokinetics of surotomycin from phase 1 single and multiple ascending dose studies in healthy volunteers. BMC Pharmacol. Toxicol..

[66] Surur A.S., Sun D. (2021). Macrocycle-Antibiotic Hybrids: A Path to Clinical Candidates. Front. Chem..

[67] Wei Y., Wu J., Chen Y., Fan K., Yu X., Li X., Zhao Y., Li Y., Lv G., Chen M. (2023). Efficacy and Safety of PL-5 (Peceleganan) Spray for Wound Infections. Ann. Surg..

[68] Khurshid Z., Najeeb S., Mali M., Moin S.F., Raza S.Q., Zohaib S., Sefat F., Zafar M.S. (2017). Histatin peptides: Pharmacological functions and their applications in dentistry. Saudi Pharm. J..

[69] Wiig M.E., Dahlin L.B., Fridén J., Hagberg L., Larsen S.E., Wiklund K., Mahlapuu M. (2014). PXL01 in Sodium Hyaluronate for Improvement of Hand Recovery after Flexor Tendon Repair Surgery: Randomized Controlled Trial. PLoS ONE.

[70] McCullough P.A., Bennett-Guerrero E., Chawla L.S., Beaver T., Mehta R.L., Molitoris B.A., Eldred A., Ball G., Lee H., Houser M.T. (2016). ABT-719 for the Prevention of Acute Kidney Injury in Patients Undergoing High-Risk Cardiac Surgery: A Randomized Phase 2b Clinical Trial. J. Am. Heart Assoc..

[71] Baker J., He X., Shi W. (2019). Precision Reengineering of the Oral Microbiome for Caries Management. Adv. Dent. Res..

[72] Greber E.K., Dawgul M. (2016). Antimicrobial Peptides Under Clinical Trials. Curr. Top. Med. Chem..

[73] Håkansson J., Ringstad L., Umerska A., Johansson J., Andersson T., Boge L., Rozenbaum R.T., Sharma P.K., Tollbäck P., Björn C. (2019). Characterization of the in vitro, ex vivo, and in vivo Efficacy of the Antimicrobial Peptide DPK-060 Used for Topical Treatment. Front. Cell. Infect. Microbiol..

[74] Khatib M.N., Shankar A.H., Kirubakaran R., Gaidhane A., Gaidhane S., Simkhada P., Syed Z.Q. (2018). Ghrelin for the management of cachexia associated with cancer. Cochrane Database Syst. Rev..

[75] Parisi K., McKenna J.A., Lowe R., Harris K.S., Shafee T., Guarino R., Lee E., van der Weerden N.L., Bleackley M.R., Anderson M.A. (2024). Hyperpolarisation of Mitochondrial Membranes Is a Critical Component of the Antifungal Mechanism of the Plant Defensin, Ppdef1. J. Fungi.

[76] Donini O., A Watkins B., Palardy J., Opal S., Sonis S., Abrams M.J., North J.R. (2010). Reduced Infection and Mucositis In Chemotherapy-Treated Animals Following Innate Defense Modulation Using a Novel Drug Candidate. Blood.

[77] Ming L., Huang J.-A. (2017). The Antibacterial Effects of Antimicrobial Peptides OP-145 against Clinically Isolated Multi-Resistant Strains. Jpn. J. Infect. Dis..

[78] Wu Y.-F., Han B.-C., Lin W.-Y., Wang S.-Y., Linn T.Y., Hsu H.W., Wen C.-C., Liu H.-Y., Chen Y.-H., Chang W.-J. (2024). Efficacy of antimicrobial peptide P113 oral health care products on the reduction of oral bacteria number and dental plaque formation in a randomized clinical assessment. J. Dent. Sci..

[79] Cheng K.-T., Wu C.-L., Yip B.-S., Chih Y.-H., Peng K.-L., Hsu S.-Y., Yu H.-Y., Cheng J.-W. (2020). The Interactions between the Antimicrobial Peptide P-113 and Living Candida albicans Cells Shed Light on Mechanisms of Antifungal Activity and Resistance. Int. J. Mol. Sci..

[80] Kowalski R.P., Romanowski E.G., Yates K.A., Mah F.S. (2016). An Independent Evaluation of a Novel Peptide Mimetic, Brilacidin (PMX30063), for Ocular Anti-Infective. J. Ocul. Pharmacol. Ther..

[81] Wang R.-R., Yang L.-M., Wang Y.-H., Pang W., Tam S.-C., Tien P., Zheng Y.-T. (2009). Sifuvirtide, a potent HIV fusion inhibitor peptide. Biochem. Biophys. Res. Commun..

[82] van der Does A.M., Bogaards S.J.P., Ravensbergen B., Beekhuizen H., van Dissel J.T., Nibbering P.H. (2010). Antimicrobial Peptide hLF1-11 Directs Granulocyte-Macrophage Colony-Stimulating Factor-Driven Monocyte Differentiation toward Macrophages with Enhanced Recognition and Clearance of Pathogens. Antimicrob. Agents Chemother..

[83] Ridyard K.E., Overhage J. (2021). The Potential of Human Peptide LL-37 as an Antimicrobial and Anti-Biofilm Agent. Antibiotics.

[84] Isaksson J., Brandsdal B.O., Engqvist M., Flaten G.E., Svendsen J.S.M., Stensen W. (2011). A Synthetic Antimicrobial Peptidomimetic (LTX 109): Stereochemical Impact on Membrane Disruption. J. Med. Chem..

[85] Chen X., Li S., Yu L., Miller A., Du L. (2019). Systematic optimization for production of the anti-MRSA antibiotics WAP-8294A in an engineered strain of *Lysobacter enzymogenes*. Microb. Biotechnol..

[86] Janec K.J., Yuan H., Jr J.E.N., Kelner R.H., Hirt C.K., Betensky R.A., Guinan E.C. (2018). rBPI 21 (opebacan) promotes rapid trilineage hematopoietic recovery in a murine model of high-dose total body irradiation. Am. J. Hematol..

[87] Schneider T., Gries K., Josten M., Wiedemann I., Pelzer S., Labischinski H., Sahl H.-G. (2009). The Lipopeptide Antibiotic Friulimicin B Inhibits Cell Wall Biosynthesis through Complex Formation with Bactoprenol Phosphate. Antimicrob. Agents Chemother..

[88] Scott M.G., Dullaghan E., Mookherjee N., Glavas N., Waldbrook M., Thompson A., Wang A., Lee K., Doria S., Hamill P. (2007). An anti-infective peptide that selectively modulates the innate immune response. Nat. Biotechnol..

[89] Boakes S., Dawson M.J. (2014). Discovery and Development of NVB302, a Semisynthetic Antibiotic for Treatment of *Clostridium difficile* Infection. Natural Products.

[90] Xiong Y.Q., Hady W.A., Deslandes A., Rey A., Fraisse L., Kristensen H.-H., Yeaman M.R., Bayer A.S. (2011). Efficacy of NZ2114, a Novel Plectasin-Derived Cationic Antimicrobial Peptide Antibiotic, in Experimental Endocarditis Due to Methicillin-Resistant Staphylococcus aureus. Antimicrob. Agents Chemother..

[91] Iwasaki M., Akiba Y., Kaunitz J.D. (2019). Recent advances in vasoactive intestinal peptide physiology and pathophysiology: Focus on the gastrointestinal system. F1000Research.

[92] Zhang Q.-Y., Yan Z.-B., Meng Y.-M., Hong X.-Y., Shao G., Ma J.-J., Cheng X.-R., Liu J., Kang J., Fu C.-Y. (2021). Antimicrobial peptides: Mechanism of action, activity and clinical potential. Mil. Med. Res..

[93] Pimchan T., Tian F., Thumanu K., Rodtong S., Yongsawatdigul J. (2023). Isolation, identification, and mode of action of antibacterial peptides derived from egg yolk hydrolysate. Poult. Sci..

[94] Walkenhorst W.F., Klein J.W., Vo P., Wimley W.C. (2013). pH Dependence of Microbe Sterilization by Cationic Antimicrobial Peptides. Antimicrob. Agents Chemother..

[95] Mahlapuu M., Björn C., Ekblom J. (2020). Antimicrobial peptides as therapeutic agents: Opportunities and challenges. Crit. Rev. Biotechnol..

[96] Chen N., Jiang C. (2023). Antimicrobial peptides: Structure, mechanism, and modification. Eur. J. Med. Chem..

[97] Giuliani A., Pirri G., Nicoletto S. (2007). Antimicrobial peptides: An overview of a promising class of therapeutics. Open Life Sci..

[98] Shai Y. (2002). Mode of action of membrane active antimicrobial peptides. Pept. Sci..

[99] Starke L.J., Allolio C., Hub J.S. (2025). How pore formation in complex biological membranes is governed by lipid composition, mechanics, and lateral sorting. PNAS Nexus.

[100] Beck K., Nandy J., Hoernke M. (2025). Strong Membrane Permeabilization Activity Can Reduce Selectivity of Cyclic Antimicrobial Peptides. J. Phys. Chem. B.

[101] Li J., Lu X., Ma W., Chen Z., Sun S., Wang Q., Yuan B., Yang K. (2021). Cholesterols Work as a Molecular Regulator of the Antimicrobial Peptide-Membrane Interactions. Front. Mol. Biosci..

[102] Espeche J.C., Martínez M., Maturana P., Cutró A., Semorile L., Maffia P.C., Hollmann A. (2020). Unravelling the mechanism of action of “de novo” designed peptide P1 with model membranes and gram-positive and gram-negative bacteria. Arch. Biochem. Biophys..

[103] Branco L.A.C., Souza P.F.N., Neto N.A.S., Aguiar T.K.B., Silva A.F.B., Carneiro R.F., Nagano C.S., Mesquita F.P., Lima L.B., Freitas C.D.T. (2022). New Insights into the Mechanism of Antibacterial Action of Synthetic Peptide Mo-CBP3-PepI against Klebsiella pneumoniae. Antibiotics.

[104] Yang L., Harroun T.A., Weiss T.M., Ding L., Huang H.W. (2001). Barrel-Stave Model or Toroidal Model? A Case Study on Melittin Pores. Biophys. J..

[105] Bechinger B., Lohner K. (2006). Detergent-like actions of linear amphipathic cationic antimicrobial peptides. Biochim. Et Biophys. Acta (BBA) Biomembr..

[106] Gazit E., Miller I.R., Biggin P.C., Sansom M.S., Shai Y. (1996). Structure and Orientation of the Mammalian Antibacterial Peptide Cecropin P1 within Phospholipid Membranes. J. Mol. Biol..

[107] Yasir M., Dutta D., Willcox M.D.P. (2019). Comparative mode of action of the antimicrobial peptide melimine and its derivative Mel4 against Pseudomonas aeruginosa. Sci. Rep..

[108] Lin Y.-M., Wu S.-J., Chang T.-W., Wang C.-F., Suen C.-S., Hwang M.-J., Chang M.D.-T., Chen Y.-T., Liao Y.-D. (2010). Outer Membrane Protein I of Pseudomonas aeruginosa Is a Target of Cationic Antimicrobial Peptide/Protein. J. Biol. Chem..

[109] Chu H., Pazgier M., Jung G., Nuccio S.-P., Castillo P.A., de Jong M.F., Winter M.G., Winter S.E., Wehkamp J., Shen B. (2012). Human α-Defensin 6 Promotes Mucosal Innate Immunity Through Self-Assembled Peptide Nanonets. Science.

[110] Riciluca K.C.T., Oliveira U.C., Mendonça R.Z., Bozelli Junior J.C., Schreier S., da Silva Junior P.I. (2021). Rondonin: Antimicrobial properties and mechanism of action. FEBS Open Bio.

[111] Lima P.G., Souza P.F., Freitas C.D., Bezerra L.P., Neto N.A., Silva A.F., Oliveira J.T., Sousa D.O. (2021). Synthetic peptides against Trichophyton mentagrophytes and T. rubrum: Mechanisms of action and efficiency compared to griseofulvin and itraconazole. Life Sci..

[112] Lopes F.E., da Costa H.P., Souza P.F., Oliveira J.P., Ramos M.V., Freire J.E., Jucá T.L., Freitas C.D. (2019). Peptide from thaumatin plant protein exhibits selective anticandidal activity by inducing apoptosis via membrane receptor. Phytochemistry.

[113] Skalickova S., Heger Z., Krejcova L., Pekarik V., Bastl K., Janda J., Kostolansky F., Vareckova E., Zitka O., Adam V. (2015). Perspective of Use of Antiviral Peptides against Influenza Virus. Viruses.

[114] Hoffmann A.R., Guha S., Wu E., Ghimire J., Wang Y., He J., Garry R.F., Wimley W.C. (2020). Broad-Spectrum Antiviral Entry Inhibition by Interfacially Active Peptides. J. Virol..

[115] Jackson J.W., Hancock T.J., Dogra P., Patel R., Arav-Boger R., Williams A.D., Kennel S.J., Wall J.S., Sparer T.E. (2019). Anticytomegalovirus Peptides Point to New Insights for CMV Entry Mechanisms and the Limitations of *In Vitro* Screenings. mSphere.

[116] Chao L., Lu L., Yang H., Zhu Y., Li Y., Wang Q., Yu X., Jiang S., Chen Y.-H. (2013). Identification of a Human Protein-Derived HIV-1 Fusion Inhibitor Targeting the gp41 Fusion Core Structure. PLoS ONE.

[117] Lin D., Li F., Wu Q., Xie X., Wu W., Wu J., Chen Q., Liu S., He J. (2016). A “building block” approach to the new influenza A virus entry inhibitors with reduced cellular toxicities. Sci. Rep..

[118] Hajigha M.N., Hajikhani B., Vaezjalali M., Kafil H.S., Anari R.K., Goudarzi M. (2024). Antiviral and antibacterial peptides: Mechanisms of action. Heliyon.

[119] Anunthawan T., de la Fuente-Núñez C., Hancock R.E., Klaynongsruang S. (2015). Cationic amphipathic peptides KT2 and RT2 are taken up into bacterial cells and kill planktonic and biofilm bacteria. Biochim. Et Biophys. Acta (BBA) Biomembr..

[120] Sneideris T., Erkamp N.A., Ausserwöger H., Saar K.L., Welsh T.J., Qian D., Katsuya-Gaviria K., Johncock M.L.L.Y., Krainer G., Borodavka A. (2023). Targeting nucleic acid phase transitions as a mechanism of action for antimicrobial peptides. Nat. Commun..

[121] Park C.B., Kim H.S., Kim S.C. (1998). Mechanism of Action of the Antimicrobial Peptide Buforin II: Buforin II Kills Microorganisms by Penetrating the Cell Membrane and Inhibiting Cellular Functions. Biochem. Biophys. Res. Commun..

[122] Li L., Li J., Yu X., Cao R., Hong M., Xu Z., Lu J.R., Wang Y., Zhu H. (2023). Antimicrobial peptides fight against Pseudomonas aeruginosa at a sub-inhibitory concentration via anti-QS pathway. Bioorganic Chem..

[123] Chen P., Zhang T., Li C., Praveen P., Parisi K., Beh C., Ding S., Wade J.D., Hong Y., Li S. (2025). Aggregation-prone antimicrobial peptides target gram-negative bacterial nucleic acids and protein synthesis. Acta Biomater..

[124] Zhang X., Yang J., Suo H., Tan J., Zhang Y., Song J. (2023). Identification and molecular mechanism of action of antibacterial peptides from Flavourzyme-hydrolyzed yak casein against Staphylococcus aureus. J. Dairy Sci..

[125] Tang Y., Yang C., Zhao J., Heng H., Peng M., Sun L., Dai L., Chan E.W.-C., Chen S. (2025). LTX-315 is a novel broad-spectrum antimicrobial peptide against clinical multidrug-resistant bacteria. J. Adv. Res..

[126] Choi H., Yang Z., Weisshaar J.C. (2017). Oxidative stress induced in E. coli by the human antimicrobial peptide LL-37. PLOS Pathog..

[127] Bermúdez-Puga S., Dias M., de Oliveira T.F., Mendonça C.M.N., de Almeida S.R.Y., Rozas E.E., Nascimento C.A.O.D., Mendes M.A., Azevedo P.O.D.S.d., Almeida J.R. (2023). Dual antibacterial mechanism of [K4K15]CZS-1 against Salmonella Typhimurium: A membrane active and intracellular-targeting antimicrobial peptide. Front. Microbiol..

[128] Chadha S. (2023). Combating fungal phytopathogens with human salivary antimicrobial peptide histatin 5 through a multi-target mechanism. World J. Microbiol. Biotechnol..

[129] Moghaddam M.-R.B., Gross T., Becker A., Vilcinskas A., Rahnamaeian M. (2017). The selective antifungal activity of Drosophila melanogaster metchnikowin reflects the species-dependent inhibition of succinate–coenzyme Q reductase. Sci. Rep..

[130] Maurya I.K., Thota C.K., Sharma J., Tupe S.G., Chaudhary P., Singh M.K., Thakur I.S., Deshpande M., Prasad R., Chauhan V.S. (2013). Mechanism of action of novel synthetic dodecapeptides against Candida albicans. Biochim. Et Biophys. Acta (BBA) Gen. Subj..

[131] Di Marino S., Scrima M., Grimaldi M., D’errico G., Vitiello G., Sanguinetti M., De Rosa M., Soriente A., Novellino E., D’ursi A.M. (2012). Antifungal peptides at membrane interaction. Eur. J. Med. Chem..

[132] Mookherjee N., Anderson M.A., Haagsman H.P., Davidson D.J. (2020). Antimicrobial host defence peptides: Functions and clinical potential. Nat. Rev. Drug Discov..

[133] Chatterjee D., Sivashanmugam K. (2024). Immunomodulatory peptides: New therapeutic horizons for emerging and re-emerging infectious diseases. Front. Microbiol..

[134] Scott M.G., Davidson D.J., Gold M.R., Bowdish D., Hancock R.E.W. (2002). The Human Antimicrobial Peptide LL-37 Is a Multifunctional Modulator of Innate Immune Responses. J. Immunol..

[135] Fjell C.D., Hiss J.A., Hancock R.E.W., Schneider G. (2012). Designing antimicrobial peptides: Form follows function. Nat. Rev. Drug Discov..

[136] Dong N., Wang C., Zhang T., Zhang L., Xue C., Feng X., Bi C., Shan A. (2019). Bioactivity and Bactericidal Mechanism of Histidine-Rich β-Hairpin Peptide Against Gram-Negative Bacteria. Int. J. Mol. Sci..

[137] de Oliveira K.B.S., Leite M.L., Melo N.T.M., Lima L.F., Barbosa T.C.Q., Carmo N.L., Melo D.A.B., Paes H.C., Franco O.L. (2024). Antimicrobial Peptide Delivery Systems as Promising Tools Against Resistant Bacterial Infections. Antibiotics.

[138] Bruno B.J., Miller G.D., Lim C.S. (2013). Basics and Recent Advances in Peptide and Protein Drug Delivery. Ther. Deliv..

[139] Katsila T., Siskos A.P., Tamvakopoulos C. (2012). Peptide and protein drugs: The study of their metabolism and catabolism by mass spectrometry. Mass Spectrom. Rev..

[140] Leuthner K.D., Yuen A., Mao Y., Rahbar A. (2015). Dalbavancin (BI-387) for the treatment of complicated skin and skin structure infection. Expert Rev. Anti-Infect. Ther..

[141] Cortes-Penfield N., Oliver N.T., Hunter A., Rodriguez-Barradas M. (2018). Daptomycin and combination daptomycin-ceftaroline as salvage therapy for persistent methicillin-resistant *Staphylococcus aureus* bacteremia. Infect. Dis..

[142] Kapić E., Becić F., Zvizdić S. (2005). Enfuvirtide, mechanism of action and pharmacological properties. Med. Arh..

[143] Pavithrra G., Rajasekaran R. (2019). Identification of Effective Dimeric Gramicidin-D Peptide as Antimicrobial Therapeutics over Drug Resistance: In-Silico Approach. Interdiscip. Sci. Comput. Life Sci..

[144] Vater J., Stein T.H. (1999). Structure, Function, and Biosynthesis of Gramicidin S Synthetase. Comprehensive Natural Products Chemistry.

[145] Greig S.L. (2016). Obiltoxaximab: First Global Approval. Drugs.

[146] Kaasch A.J., Seifert H. (2016). Oritavancin: A Long-Acting Antibacterial Lipoglycopeptide. Future Microbiol..

[147] Mazur N.I., Löwensteyn Y.N., Terstappen J., Leusen J., Schobben F., Cianci D., van de Ven P.M., Nierkens S., Bont L.J., Nibbelke E.E. (2023). Daily intranasal palivizumab to prevent respiratory syncytial virus infection in healthy preterm infants: A phase 1/2b randomized placebo-controlled trial. eClinicalMedicine.

[148] Avedissian S.N., Liu J., Rhodes N.J., Lee A., Pais G.M., Hauser A.R., Scheetz M.H. (2019). A Review of the Clinical Pharmacokinetics of Polymyxin B. Antibiotics.

[149] Soon R.L., Nation R.L., Cockram S., Moffatt J.H., Harper M., Ben Adler B., Boyce J.D., Larson I., Li J. (2010). Different surface charge of colistin-susceptible and -resistant Acinetobacter baumannii cells measured with zeta potential as a function of growth phase and colistin treatment. J. Antimicrob. Chemother..

[150] Zhanel G.G., Calic D., Schweizer F., Zelenitsky S., Adam H., Lagacé-Wiens P.R.S., Rubinstein E., Gin A.S., Hoban D.J., Karlowsky J.A. (2010). New Lipoglycopeptides. Drugs.

[151] Romani L., Bistoni F., Gaziano R., Bozza S., Montagnoli C., Perruccio K., Pitzurra L., Bellocchio S., Velardi A., Rasi G. (2004). Thymosin α 1 activates dendritic cells for antifungal Th1 resistance through Toll-like receptor signaling. Blood.

[152] Vosloo J.A., Rautenbach M. (2020). Following tyrothricin peptide production by Brevibacillus parabrevis with electrospray mass spectrometry. Biochimie.

[153] Gramenzi A., Cursaro C., Andreone P., Bernardi M. (1998). Thymalfasin. BioDrugs.

[154] Rybak M.J. (2006). The Pharmacokinetic and Pharmacodynamic Properties of Vancomycin. Clin. Infect. Dis..

[155] Knox C., Wilson M., Klinger C.M., Franklin M., Oler E., Wilson A., Pon A., Cox J., Chin N.E., A Strawbridge S. (2024). DrugBank 6.0: The DrugBank Knowledgebase for 2024. Nucleic Acids Res..

[156] Kim S., Chen J., Cheng T., Gindulyte A., He J., He S., Li Q., Shoemaker B., Thiessen P.A., Yu B. (2025). PubChem 2025 update. Nucleic Acids Res..

[157] Ooijevaar R.E., van Beurden Y.H., Terveer E.M., Goorhuis A., Bauer M.P., Keller J.J., Mulder C.J.J., Kuijper E.J. (2018). Update of treatment algorithms for Clostridium difficile infection. Clin. Microbiol. Infect..

[158] Rosson E., Lux F., David L., Godfrin Y., Tillement O., Thomas E. (2025). Focus on therapeutic peptides and their delivery. Int. J. Pharm..

[159] Greco I., Molchanova N., Holmedal E., Jenssen H., Hummel B.D., Watts J.L., Håkansson J., Hansen P.R., Svenson J. (2020). Correlation between hemolytic activity, cytotoxicity and systemic in vivo toxicity of synthetic antimicrobial peptides. Sci. Rep..

[160] Prasad N.K., Seiple I.B., Cirz R.T., Rosenberg O.S. (2022). Leaks in the Pipeline: A Failure Analysis of Gram-Negative Antibiotic Development from 2010 to 2020. Antimicrob. Agents Chemother..

[161] Dale G.E., Halabi A., Petersen-Sylla M., Wach A., Zwingelstein C. (2018). Pharmacokinetics, Tolerability, and Safety of Murepavadin, a Novel Antipseudomonal Antibiotic, in Subjects with Mild, Moderate, or Severe Renal Function Impairment. Antimicrob. Agents Chemother..

[162] Fadaka A.O., Sibuyi N.R.S., Madiehe A.M., Meyer M. (2021). Nanotechnology-Based Delivery Systems for Antimicrobial Peptides. Pharmaceutics.

[163] Haddadzadegan S., Dorkoosh F., Bernkop-Schnürch A. (2022). Oral delivery of therapeutic peptides and proteins: Technology landscape of lipid-based nanocarriers. Adv. Drug Deliv. Rev..

[164] Tang T., Chen Y., Zhao Z., Bai Q., Leisner J.J., Liu T. (2024). Nisin-loaded chitosan/sodium alginate microspheres enhance the antimicrobial efficacy of nisin against *Staphylococcus aureus*. J. Appl. Microbiol..

[165] Hayes H.C., Luk L.Y.P., Tsai Y.-H. (2021). Approaches for peptide and protein cyclisation. Org. Biomol. Chem..

[166] Bellavita R., Braccia S., Galdiero S., Falanga A. (2023). Glycosylation and Lipidation Strategies: Approaches for Improving Antimicrobial Peptide Efficacy. Pharmaceuticals.

[167] Erak M., Bellmann-Sickert K., Els-Heindl S., Beck-Sickinger A.G. (2018). Peptide chemistry toolbox—Transforming natural peptides into peptide therapeutics. Bioorganic Med. Chem..

[168] Biondi S., Chugunova E., Panunzio M., Atta-ur-Rahman (2016). Chapter 8—From Natural Products to Drugs: Glyco- and Lipoglycopeptides, a New Generation of Potent Cell Wall Biosynthesis Inhibitors. Studies in Natural Products Chemistry.

[169] Yan J., Cai J., Zhang B., Wang Y., Wong D.F., Siu S.W.I. (2022). Recent Progress in the Discovery and Design of Antimicrobial Peptides Using Traditional Machine Learning and Deep Learning. Antibiotics.

[170] Wang Y., Ding Y., Wen H., Lin Y., Hu Y., Zhang Y., Xia Q., Lin Z. (2012). QSAR Modeling and Design of Cationic Antimicrobial Peptides Based on Structural Properties of Amino Acids. Comb. Chem. High Throughput Screen..

[171] Nedyalkova M., Paluch A.S., Vecini D.P., Lattuada M. (2024). Progress and future of the computational design of antimicrobial peptides (AMPs): Bio-inspired functional molecules. Digit. Discov..

[172] Cardoso M.H., Orozco R.M.Q., Rezende S.B., Rodrigues G., Oshiro K.G.N., Cândido E.D.S., Franco O.L. (2020). Computer-Aided Design of Antimicrobial Peptides: Are We Generating Effective Drug Candidates?. Front. Microbiol..

[173] Ramazi S., Mohammadi N., Allahverdi A., Khalili E., Abdolmaleki P. (2022). A review on antimicrobial peptides databases and the computational tools. Database.

[174] Agüero-Chapin G., Galpert-Cañizares D., Domínguez-Pérez D., Marrero-Ponce Y., Pérez-Machado G., Teijeira M., Antunes A. (2022). Emerging Computational Approaches for Antimicrobial Peptide Discovery. Antibiotics.

[175] Mwangi J., Kamau P.M., Thuku R.C., Lai R. (2023). Design Methods for An.timicrobial Peptides with Improved Performance. Zool. Res..

[176] Brizuela C.A., Liu G., Stokes J.M., de la Fuente-Nunez C. (2025). Methods for Antimicrobial Peptides: Progress and Challenges. Microb. Biotechnol..

[177] Zhou X., Liu G., Cao S., Lv J. (2025). Deep Learning for Antimicrobial Peptides: Computational Models and Databases. J. Chem. Inf. Model..

[178] Mirdita M., Schütze K., Moriwaki Y., Heo L., Ovchinnikov S., Steinegger M. (2022). ColabFold: Making protein folding accessible to all. Nat. Methods.

[179] Baek M., DiMaio F., Anishchenko I., Dauparas J., OvchinBnikov S., Lee G.R., Wang J., Cong Q., Kinch L.N., Schaeffer R.D. (2021). Accurate prediction of protein structures and interactions using a three-track neural network. Science.

[180] Humphreys I.R., Pei J., Baek M., Krishnakumar A., Anishchenko I., Ovchinnikov S., Zhang J., Ness T.J., Banjade S., Bagde S.R. (2021). Computed structures of core eukaryotic protein complexes. Science.

[181] Trott O., Olson A.J. (2010). AutoDock Vina: Improving the speed and accuracy of docking with a new scoring function, efficient optimization, and multithreading. J. Comput. Chem..

[182] Giulini M., Reys V., Teixeira J.M.C., Jiménez-García B., Honorato R.V., Kravchenko A., Xu X., Versini R., Engel A., Verhoeven S. (2025). HADDOCK3: A modular and versatile platform for integrative modelling of biomolecular complexes. bioRxiv.

[183] Kurcinski M., Jamroz M., Blaszczyk M., Kolinski A., Kmiecik S. (2015). CABS-dock web server for the flexible docking of peptides to proteins without prior knowledge of the binding site. Nucleic Acids Res..

[184] Abraham M.J., Murtola T., Schulz R., Páll S., Smith J.C., Hess B., Lindahl E. (2015). GROMACS: High performance molecular simulations through multi-level parallelism from laptops to supercomputers. SoftwareX.

[185] Huang J., MacKerell A.D. (2013). CHARMM36 all-atom additive protein force field: Validation based on comparison to NMR data. J. Comput. Chem..

[186] Marrink S.J., Risselada H.J., Yefimov S., Tieleman D.P., de Vries A.H. (2007). The MARTINI Force Field: Coarse Grained Model for Biomolecular Simulations. J. Phys. Chem. B.

[187] Fu H., Cao Z., Li M., Wang S. (2020). ACEP: Improving antimicrobial peptides recognition through automatic feature fusion and amino acid embedding. BMC Genom..

[188] Ramos-Martín F., Annaval T., Buchoux S., Sarazin C., D’aMelio N. (2019). ADAPTABLE: A comprehensive web platform of antimicrobial peptides tailored to the user’s research. Life Sci. Alliance.

[189] Fang Y., Xu F., Wei L., Jiang Y., Chen J., Wei L., Wei D.-Q. (2023). AFP-MFL: Accurate identification of antifungal peptides using multi-view feature learning. Brief. Bioinform..

[190] Veltri D., Kamath U., Shehu A. (2017). Improving Recognition of Antimicrobial Peptides and Target Selectivity through Machine Learning and Genetic Programming. IEEE/ACM Trans. Comput. Biol. Bioinform..

[191] Torrent M., Nogués V.M., Boix E. (2009). A theoretical approach to spot active regions in antimicrobial proteins. BMC Bioinform..

[192] Torrent M., Di Tommaso P., Pulido D., Nogués M.V., Notredame C., Boix E., Andreu D. (2012). AMPA: An automated web server for prediction of protein antimicrobial regions. Bioinformatics.

[193] Salem M., Arshadi A.K., Yuan J.S. (2022). AMPDeep: Hemolytic activity prediction of antimicrobial peptides using transfer learning. BMC Bioinform..

[194] Lawrence T.J., Carper D.L., Spangler M.K., A Carrell A., A Rush T., Minter S.J., Weston D.J., Labbé J.L. (2021). amPEPpy 1.0: A portable and accurate antimicrobial peptide prediction tool. Bioinformatics.

[195] Burdukiewicz M., Sidorczuk K., Rafacz D., Pietluch F., Chilimoniuk J., Rödiger S., Gagat P. (2020). Proteomic Screening for Prediction and Design of Antimicrobial Peptides with AmpGram. Int. J. Mol. Sci..

[196] Cai J., Yan J., Un C., Wang Y., Campbell-Valois F.-X., Siu S.W.I. (2025). BERT-AmPEP60: A BERT-Based Transfer Learning Approach to Predict the Minimum Inhibitory Concentrations of Antimicrobial Peptides for *Escherichia coli* and *Staphylococcus aureus*. J. Chem. Inf. Model..

[197] Porto W.F., Pires Á.S., Franco O.L. (2012). CS-AMPPred: An Updated SVM Model for Antimicrobial Activity Prediction in Cysteine-Stabilized Peptides. PLoS ONE.

[198] Slavokhotova A.A., Shelenkov A.A., Rogozhin E.A. (2024). Computational Prediction and Structural Analysis of α-Hairpinins, a Ubiquitous Family of Antimicrobial Peptides, Using the Cysmotif Searcher Pipeline. Antibiotics.

[199] Azim S.M., Sharma A., Noshadi I., Shatabda S., Dehzangi I. (2021). DeepAmp: A Convolutional Neural Network Based Tool for Predicting Protein AMPylation Sites from Binary Profile Representation. Res. Sq..

[200] Xu J., Li F., Li C., Guo X., Landersdorfer C., Shen H.-H., Peleg A.Y., Li J., Imoto S., Yao J. (2023). iAMPCN: A deep-learning approach for identifying antimicrobial peptides and their functional activities. Brief. Bioinform..

[201] Tang W., Dai R., Yan W., Zhang W., Bin Y., Xia E., Xia J. (2022). Identifying multi-functional bioactive peptide functions using multi-label deep learning. Brief. Bioinform..

[202] Tang W., Dai R., Yan W., Zhang W., Bin Y., Xia E., Xia J. (2021). Comprehensive assessment of machine learning-based methods for predicting antimicrobial peptides. Brief. Bioinform..

[203] Bajiya N., Choudhury S., Dhall A., Raghava G.P.S. (2024). AntiBP3: A Method for Predicting Antibacterial Peptides against Gram-Positive/Negative/Variable Bacteria. Antibiotics.

[204] Porto W.F., Fensterseifer I.C., Ribeiro S.M., Franco O.L. (2018). Joker: An algorithm to insert patterns into sequences for designing antimicrobial peptides. Biochim. Et Biophys. Acta (BBA) Gen. Subj..

[205] Müller A.T., Gabernet G., A Hiss J., Schneider G. (2017). modlAMP: Python for antimicrobial peptides. Bioinformatics.

[206] Varadi M., Bertoni D., Magana P., Paramval U., Pidruchna I., Radhakrishnan M., Tsenkov M., Nair S., Mirdita M., Yeo J. (2024). AlphaFold Protein Structure Database in 2024: Providing structure coverage for over 214 million protein sequences. Nucleic Acids Res..

[207] Jumper J., Evans R., Pritzel A., Green T., Figurnov M., Ronneberger O., Tunyasuvunakool K., Bates R., Žídek A., Potapenko A. (2021). Highly accurate protein structure prediction with AlphaFold. Nature.

[208] Zheng W., Wuyun Q., Li Y., Liu Q., Zhou X., Peng C., Zhu Y., Freddolino L., Zhang Y. (2025). Deep-learning-based single-domain and multidomain protein structure prediction with D-I-TASSER. Nat. Biotechnol..

[209] Singh H., Singh S., Singh Raghava G.P. (2019). Peptide Secondary Structure Prediction using Evolutionary Information. bioRxiv.

[210] Humphrey W., Dalke A., Schulten K. (1996). VMD: Visual molecular dynamics. J. Mol. Graph..

[211] Phillips J.C., Braun R., Wang W., Gumbart J., Tajkhorshid E., Villa E., Chipot C., Skeel R.D., Kalé L., Schulten K. (2005). Scalable molecular dynamics with NAMD. J. Comput. Chem..

[212] Fang Y., Ma Y., Yu K., Dong J., Zeng W. (2024). Integrated computational approaches for advancing antimicrobial peptide development. Trends Pharmacol. Sci..

[213] Lee H.T., Lee C.C., Yang J.R., Lai J.Z., Chang K.Y. (2015). A Large-Scale Structural Classification of Antimicrobial Peptides. BioMed Res. Int..

[214] Alcock B.P., Huynh W., Chalil R., Smith K.W., Raphenya A.R., A Wlodarski M., Edalatmand A., Petkau A., A Syed S., Tsang K.K. (2023). CARD 2023: Expanded curation, support for machine learning, and resistome prediction at the Comprehensive Antibiotic Resistance Database. Nucleic Acids Res..

[215] Novković M., Simunić J., Bojović V., Tossi A., Juretić D. (2012). DADP: The database of anuran defense peptides. Bioinformatics.

[216] Yao L., Guan J., Xie P., Chung C.-R., Zhao Z., Dong D., Guo Y., Zhang W., Deng J., Pang Y. (2025). dbAMP 3.0: Updated resource of antimicrobial activity and structural annotation of peptides in the post-pandemic era. Nucleic Acids Res..

[217] Gautam A., Chaudhary K., Singh S., Joshi A., Anand P., Tuknait A., Raghava G.P.S. (2014). Hemolytik: A database of experimentally determined hemolytic and non-hemolytic peptides. Nucleic Acids Res..

[218] Gómez E.A., Giraldo P., Orduz S. (2017). InverPep: A database of invertebrate antimicrobial peptides. J. Glob. Antimicrob. Resist..

[219] Bonin N., Doster E., Worley H., Pinnell L.J., E Bravo J., Ferm P., Marini S., Prosperi M., Noyes N., Morley P.S. (2022). MEGARes and AMR++, v3.0: An updated comprehensive database of antimicrobial resistance determinants and an improved software pipeline for classification using high-throughput sequencing. Nucleic Acids Res..

[220] D’aLoisio V., Dognini P., Hutcheon G.A., Coxon C.R. (2021). PepTherDia: Database and structural composition analysis of approved peptide therapeutics and diagnostics. Drug Discov. Today.

[221] Usmani S.S., Bedi G., Samuel J.S., Singh S., Kalra S., Kumar P., Ahuja A.A., Sharma M., Gautam A., Raghava G.P.S. (2017). THPdb: Database of FDA-approved peptide and protein therapeutics. PLoS ONE.

[222] Piotto S.P., Sessa L., Concilio S., Iannelli P. (2012). YADAMP: Yet another database of antimicrobial peptides. Int. J. Antimicrob. Agents.

[223] Fabbri L.P., Cavallero A., Vidotto F., Gabriele M. (2024). Bioactive Peptides from Fermented Foods: Production Approaches, Sources, and Potential Health Benefits. Foods.

[224] Bisht V., Das B., Hussain A., Kumar V., Navani N.K. (2024). Understanding of probiotic origin antimicrobial peptides: A sustainable approach ensuring food safety. NPJ Sci. Food.

[225] de la Lastra J.M.P., González-Acosta S., Otazo-Pérez A., Asensio-Calavia P., Rodríguez-Borges V.M. (2025). Antimicrobial Peptides for Food Protection: Leveraging Edible Mushrooms and Nano-Innovation. Dietetics.

[226] Kumar L., Tyagi P., Lucia L., Pal L. (2025). Innovations in Edible Packaging Films, Coatings, and Antimicrobial Agents for Applications in Food Industry. Compr. Rev. Food Sci. Food Saf..

[227] Choi D., Bedale W., Chetty S., Yu J. (2024). Comprehensive review of clean-label antimicrobials used in dairy products. Compr. Rev. Food Sci. Food Saf..

[228] Min K.H., Kim K.H., Ki M.-R., Pack S.P. (2024). Antimicrobial Peptides and Their Biomedical Applications: A Review. Antibiotics.

[229] Sengkhui S., Klubthawee N., Aunpad R. (2023). A novel designed membrane-active peptide for the control of foodborne Salmonella enterica serovar Typhimurium. Sci. Rep..

[230] Wang L., Dekker M., Heising J., Zhao L., Fogliano V. (2024). Food matrix design can influence the antimicrobial activity in the food systems: A narrative review. Crit. Rev. Food Sci. Nutr..

[231] Chu Z., Wang H., Dong B. (2024). Research on Food Preservation Based on Antibacterial Technology: Progress and Future Prospects. Molecules.

[232] Swann M. (1969). Report/Joint Committee on the use of Antibiotics in Animal Husbandry and Veterinary Medicine.

[233] Vercelli C., Gambino G., Amadori M., Re G. (2022). Implications of Veterinary Medicine in the comprehension and stewardship of antimicrobial resistance phenomenon. From the origin till nowadays. Vet. Anim. Sci..

[234] Vittecoq M., Godreuil S., Prugnolle F., Durand P., Brazier L., Renaud N., Arnal A., Aberkane S., Jean-Pierre H., Gauthier-Clerc M. (2016). Antimicrobial resistance in wildlife. J. Appl. Ecol..

[235] Valdez-Miramontes C., De Haro-Acosta J., Aréchiga-Flores C., Verdiguel-Fernández L., Rivas-Santiago B. (2021). Antimicrobial peptides in domestic animals and their applications in veterinary medicine. Peptides.

[236] Tai H.-M., Huang H.-N., Tsai T.-Y., You M.-F., Wu H.-Y., Rajanbabu V., Chang H.-Y., Pan C.-Y., Chen J.-Y. (2020). Dietary supplementation of recombinant antimicrobial peptide Epinephelus lanceolatus piscidin improves growth performance and immune response in Gallus gallus domesticus. PLoS ONE.

[237] Tang X.-S., Shao H., Li T.-J., Tang Z.-R., Huang R.-L., Wang S.-P., Kong X.-F., Wu X., Yin Y.-L. (2012). Dietary Supplementation with Bovine Lactoferrampin–Lactoferricin Produced by Pichia pastoris Fed-batch Fermentation Affects Intestinal Microflora in Weaned Piglets. Appl. Biochem. Biotechnol..

[238] Yoon J.H., Ingale S.L., Kim J.S., Kim K.H., Lohakare J., Park Y.K., Park J.C., Kwon I.K., Chae B.J. (2013). Effects of dietary supplementation with antimicrobial peptide-P5 on growth performance, apparent total tract digestibility, faecal and intestinal microflora and intestinal morphology of weanling pigs. J. Sci. Food Agric..

[239] Wang L., Zhao X., Zhu C., Zhao Y., Liu S., Xia X., Liu X., Zhang H., Xu Y., Hang B. (2020). The antimicrobial peptide MPX kills Actinobacillus pleuropneumoniae and reduces its pathogenicity in mice. Vet. Microbiol..

[240] Xu Y., Wang Q., Dong M., Song H., Hang B., Sun Y., Zhang H., Hu J. (2023). Evaluation of the efficacy of the antimicrobial peptide HJH-3 in chickens infected with *Salmonella* Pullorum. Front. Microbiol..

[241] Bruhn O., Grötzinger J., Cascorbi I., Jung S. (2011). Antimicrobial peptides and proteins of the horse—Insights into a well-armed organism. Vet. Res..

[242] Shen H., Li Y., Pi Q., Tian J., Xu X., Huang Z., Huang J., Pian C., Mao S. (2025). Unveiling novel antimicrobial peptides from the ruminant gastrointestinal microbiomes: A deep learning-driven approach yields an anti-MRSA candidate. J. Adv. Res..

